# Predicting neurosurgical outcomes in focal epilepsy patients using computational modelling

**DOI:** 10.1093/brain/aww299

**Published:** 2016-12-30

**Authors:** Nishant Sinha, Justin Dauwels, Marcus Kaiser, Sydney S Cash, M Brandon Westover, Yujiang Wang, Peter N Taylor

**Affiliations:** 1 School of Electrical and Electronic Engineering, Nanyang Technological University, Singapore; 2 Interdisciplinary Computing and Complex BioSystems (ICOS) Research Group, School of Computing Science, Newcastle University, Newcastle upon Tyne, UK; 3 Institute of Neuroscience, Faculty of Medical Science, Newcastle University, Newcastle upon Tyne, UK; 4 Massachusetts General Hospital and Harvard Medical School, Boston, MA, USA; 5 Institute of Neurology, University College London, UK

**Keywords:** epilepsy, focal seizures, computational models, intracranial EEG, surgical outcome prediction

## Abstract

**See Eissa and Schevon (doi:10.1093/aww332) for a scientific commentary on this article**.

Surgery can be a last resort for patients with intractable, medically refractory epilepsy. For many of these patients, however, there is substantial risk that the surgery will be ineffective. The prediction of who is likely to benefit from a surgical approach is crucial for being able to inform patients better, conduct principled prospective clinical trials, and ultimately tailor therapeutic approaches to these patients more effectively. Dynamical computational models, informed with patient data, can be used to make predictions and give mechanistic insight. In this study, we develop patient-specific dynamical network models of epileptogenic cortex. We infer the network connectivity matrix from non-seizure electrographic recordings of patients and use these connectivity matrices as the network structure in our model. The model simulates the dynamics of a bi-stable switch at every node in this network, meaning that every node starts in a background state, but has the ability to transit to a co-existing seizure state. Whether a transition happens in a node is partly determined by the stochastic nature of the input to the node, but also by the input the node receives from other connected nodes in the network. By conducting simulations with such a model, we can detect the average transition time for nodes in a given network, and therefore define nodes with a short transition time as highly epileptogenic. In a retrospective study, we found that in some patients the regions with high epileptogenicity in the model overlap with those identified clinically as the seizure onset zone. Moreover, it was found that the resection of these regions in the model reduces the overall likelihood of a seizure. Following removal of these regions in the model, we predicted surgical outcomes and compared these to actual patient outcomes. Our predictions were found to be 81.3% accurate on a dataset of 16 patients with intractable epilepsy. Intriguingly, in patients with unsuccessful outcomes, the proposed computational approach is able to suggest alternative resection sites. The model presented here gives mechanistic insight as to why surgery may be unsuccessful in some patients. This may aid clinicians in presurgical evaluation by providing a tool to explore various surgical options, offering complementary information to existing clinical techniques.

## Teaser

Neurosurgical treatment of focal epilepsy is highly unpredictable in cases where a lesion is not apparent by any imaging modality. Sinha *et al.* propose a computational approach to predict the outcome of a planned resection prior to surgery and investigate the pathophysiology of epileptic networks to delineate cortical areas crucial for ictogenesis.

## Introduction

Focal epilepsy is a common neurological disorder characterized by recurrent seizures together with abnormal electrographic activity in localized (focal) brain areas. Approximately 30% of patients suffering from focal seizures are refractory to medication, hence surgical intervention is considered as an alternative treatment. To determine the location of the seizure focus, presurgical evaluations are usually performed, using a combination of the history, physical exam, neuroimaging, EEG and other modalities (Rosenow and Lüders, 2001; Duncan *et al.*, 2016). In some patients these studies are insufficient and intracranial EEG is required with a focus on ictal activity as the prime marker of brain regions underlying the epilepsy. Long hospitalization times are often required for enough seizures to be captured using intracranial electrodes. If the location of the epileptic focus is considered to be identified and not eloquent, the patient undergoes surgical resection of the epileptic tissue. In cases with a clear-cut lesion seen on neuroimaging, surgery renders up to 80% of patients seizure-free (Wiebe *et al.*, 2001; Choi *et al.*, 2008; Jobst and Cascino, 2015). However, in ‘non-lesional’ cases, surgical failure rates are up to 50%, where seizures occur with a similar frequency after the surgery (Yoon *et al.*, 2003; de Tisi *et al.*, 2011). It will be beneficial to be able to predict in a patient-specific manner when surgery will not work and to suggest alternative resection sites.

One of the proposed reasons for unsuccessful surgical resections is the notion that even focal epilepsies are network diseases (Bragin *et al.*, 2000; Spencer, 2002; Kramer and Cash, 2012; Terry *et al.*, 2012; Lam *et al.*, 2016). This notion suggests that epilepsy can be considered a disease of abnormal network organization of brain areas and the connections between them. Indeed, many studies have shown alterations in structural brain networks of patients with focal epilepsies relative to controls (Bonilha *et al.*, 2012; Richardson, 2012; Diessen *et al.*, 2013; Taylor *et al.*, 2015*a*). In functional networks, areas of abnormally increased synchronization have been identified during interictal (non-seizure) periods, which appear to overlap with the seizure onset zone (Schevon *et al.*, 2007; Dauwels *et al.*, 2009; Laufs *et al.*, 2012; van Mierlo *et al.*, 2014). Such network approaches have also been successfully applied to find differences between patient groups who have differing surgical outcomes (Bonilha *et al.*, 2015; Englot *et al.*, 2015; Munsell *et al.*, 2015; Coan *et al.*, 2016). This suggests that functional and structural networks potentially contain information that could be of predictive value. However, these approaches tend to rely on the analysis of the static network structure, and largely disregard dynamical properties, which are known to be important in the generation of seizures (Taylor *et al.*, 2014*a*).

Dynamical simulations using computer models have provided mechanistic insight into how network features relate to clinical manifestations of seizures (Wendling *et al.*, 2002; Terry *et al.*, 2012; Taylor *et al.*, 2013; Jirsa *et al.*, 2014; Schmidt *et al.*, 2014). Furthermore, network modelling of seizure transitions has suggested improved classification of seizure types (Wang *et al.*, 2014), enabled the prediction of optimal stimulation protocols (Taylor *et al.*, 2015*b*) and suggested alternate seizure onset mechanisms (Lopes da Silva *et al.*, 2003; Goodfellow *et al.*, 2011; Baier *et al.*, 2012). However, only limited work has been done using dynamical network modelling approaches in the context of epilepsy surgery (Sinha *et al.*, 2014; Hutchings *et al.*, 2015; Goodfellow *et al.*, 2016).

In this retrospective study, we combine dynamical simulations and functional connectivity derived from interictal electrocorticographic (ECoG) recordings previously acquired and where the surgical outcome is known. The aim of this study is to predict surgical outcomes by simulating surgery (i.e. removal of nodes from the network) *in silico*. Finally, we use the model as a network measure to suggest alternative resection approaches for patients with poor predicted outcomes.

## Materials and methods

### Patient information and recordings

We retrospectively studied 16 patients having long-standing pharmacoresistant epilepsy who were treated at Massachusetts General Hospital (MGH), and Mayo Clinic (publicly available from IEEG portal; https://www.ieeg.org). Patients selected had seizures with focal onset and typical complex partial events, often with secondary generalization. The mean age of patients was 25.06 ± 16.42 years, and seven patients were female. These patients underwent surgical therapy with a goal of achieving seizure freedom, and the seizure focus was delineated using standard clinical techniques (e.g. ECoG, seizure recordings, MRI). The seizure onset regions were most common in the neocortical temporal lobe and in mesial temporal lobe or a mix of the two (*n* = 9). In four patients, seizures arose from frontal lobe structures and in another four patients, seizure arose from the parietal and occipital lobes.

Surgical outcome was defined as at least 1 year of post-surgical follow-up according to the ILAE surgical outcome scale. Patients were categorized in two groups: good outcome and poor outcome. Good outcome cases correspond to surgical outcome class I or II and poor outcome cases correspond to surgical outcome class III, IV or V. Based on this classification, eight patients were classified to have good post-surgical outcome and eight were classified to have poor post-surgical outcome. The clinical and demographic information of all patients in this study is summarized in Table 1.

**Table 1:** Patient information, actual surgical outcomes and retrospectively predicted outcome

Patient ID	Sex	Age^a^ onset	Age^a^ at surgery	Location of surgery	Surgical outcome (ILAE Class)	Prediction $d_{\mathrm{actual:rand}}$	Cohen’s $d_{\mathrm{actual:rand}}$	Prediction $t_{\mathrm{actual-random}}$	Escape time $t_{actual-random}$
P1	F	11–20	21–30	Temporal lobectomy	Seizure free (II)	Good	9.746	Good	38.38
P2^b^	M	1–10	1–10	Parietal corticectomy	Not seizure free (IV)	Poor	−2.504	Poor	−33.93
P3	F	11–20	41–50	Posterior temporal lesionectomy	Seizure free (I)	Good	1.729	Good	38.42
P4	F	1–10	50–60	Temporal lobectomy	Seizure free (I)	Good	0.811	Good	15.82
P5	M	1–10	11–20	Parietal corticectomy	Seizure free (I)	Poor	−0.894	Good	−7.03
P6^b^	M	51–60	51–60	Medial frontal lobectomy; amygdalohippocampectomy	Seizure free (I)	Good	5.1529	Good	106.66
P7^b^	M	11–20	11–20	Temporal lobectomy; amygdalohippocampectomy	Seizure free (I)	Poor	−1.911	Poor	−23.81
P8^b^	M	1–10	11–20	Occipital lobectomy	Seizure free (I)	Poor	−0.174	Good	−2.751
P9^b^	M	1–10	1–10	Frontal corticectomy	Seizure free (I)	Good	0.5147	Good	4.42
P10^b^	F	11–20	21–30	Temporo-occipital lobectomy	Not seizure Free (IV)	Poor	0.299	Good	4.61
P11^b^	F	1–10	31–40	Temporal lobectomy	Not seizure free (IV)	Poor	−0.322	Poor	−13.29
P12^b^	F	11–20	21–30	Temporal lobectomy; amygdalohippocampectomy	Not seizure free (V)	Poor	−0.576	Poor	−11.75
P13^b^	M	1–10	1–10	Frontal lobectomy	Not seizure free (IV)	Poor	0.038	Good	2.664
P14^b^	M	31–40	31–40	Temporal lobectomy	Not seizure free (V)	Poor	−6.005	Poor	−185.37
P15^b^	F	11–20	21–30	Temporal lobectomy	Not seizure free (V)	Poor	−4.934	Poor	−118.84
P16^b^	M	1–10	1–10	Frontal lesionectomy	Not seizure free (V)	Poor	−2.062	Poor	−8.921

a Actual age has been changed to age groups to maintain the anonymity of patients.

b Diagnosed at Mayo Clinic. More details available in Supplementary Table 7.

All recordings were performed using standard clinical recording systems with a sampling rate of 500 Hz. Two-dimensional subdural electrode arrays as well as linear arrays of electrodes (grid/strips and depth electrodes) were placed to confirm the hypothesized seizure focus, and locate epileptogenic tissue in relation to eloquent cortex, thus directing surgical treatment. The decision to implant the electrode targets and the duration of implantation were made entirely on clinical grounds with no input from this research study. For the analysis presented here we focused only on the grid and strip electrodes placed on the cortex. All data were collected conforming to ethical guidelines and under protocols monitored by the local Institutional Review Boards (IRB) according to NIH guidelines.

### Data preprocessing, functional network and ground truth resection site

We extracted a 1-h segment of interictal (non-seizure) ECoG data for each patient. The ECoG recordings used are from apparently ‘healthy’ interictal epochs only, with no obvious epileptiform activity, and recorded several hours away from any clinical seizure where possible. The data were band-pass filtered between 1 to 70 Hz, and notch filtered at 60 Hz to exclude power line interference. A common reference was used for data analysis and the reference electrode in each case was located far from the area of recording making the introduction of spurious correlation or elimination of actual correlation between cortical regions unlikely (Dauwels *et al.*, 2009). Use of a Laplacian montage appeared to give no better results, but rather have the opposite effect (Supplementary Fig. 8). The channels were not selected based on any pre-existing knowledge, except that clearly dysfunctional channels were discarded. Symmetric functional connectivity *C_ij_* between two regions of the brain *i* and *j* was computed as the average correlation (see below) of the signal recorded by the electrode contacts of those regions. The use of asymmetric functional connectivity measure gave broadly similar results (Supplementary Fig. 9).

To extract (at least approximately) the stationary aspects of ECoG data, we chose to divide the ECoG signal into consecutive 1-s segments, whereby each segment overlaps the previous segment by 0.5 s (Kramer *et al.*, 2008; Dauwels *et al.*, 2009; Antony *et al.*, 2013). The correlation was calculated within each 1-s segment. By averaging over all 1-s segments of a 1-h ECoG signal, we obtain average values of the functional connectivity for the 1-h ECoG signal. Note that all values of the correlation matrix are bounded between −1 and +1. Negative correlation values implying long range inhibitions were set to zero, as within our modelling framework and in line with previous studies (Benjamin *et al.*, 2012; Petkov *et al.*, 2014), we do not consider the contribution of long range direct inhibitory connections to the simulation of the epileptogenic effect.

The location of surgical resection (our ground truth for what was actually resected) was determined for the iEEG data after analysing the clinical reports for locating seizure focus and seizure spread using prolonged video-ECoG monitoring, surgery reports detailing resection procedures, pathology reports of resected cortical tissues, and imaging data (wherever available) showing the precise location of ECoG electrodes. Based on these reports, the electrodes contained within the site of resection were independently analysed by three of the authors (N.S., Y.W., P.T.) before arriving at a consensus. For the MGH data, the surgical resection site was determined by clinicians at MGH, independently of this study. These electrodes are shaded in black for each patient in Fig. 4 and Supplementary Fig. 1.

### Mathematical model

To investigate how the patterns of functional interactions determine the dynamics of seizure initiation, we incorporated the functional network into a dynamical model. The model dynamics are based on the hypothesis that the change in the brain state causes seizure onset and this change is driven by noise in a bi-stable system (Lopes da Silva *et al.*, 2003; Suffczynski *et al.*, 2006; Kalitzin *et al.*, 2010; Benjamin *et al.*, 2012; Taylor *et al.*, 2014*b*). Building on the aforementioned, and the methods of Terry *et al.* (2013), who suggested the use of such a model in the context of generalized epilepsy, we use a similar approach for focal seizures and surgery localization. Our objective is to study the role of network structure in transitions between non-seizure and seizure states. Therefore, in this modelling framework, we consider the cortical region under each ECoG electrode as a network node. Individually each node, in a bi-stable setting of the model parameters, produces a simulated time series with a resting state and episodes of pathological high amplitude oscillations. These oscillations are identified with ictal (seizure) dynamics, whereas the resting state is identified with interictal (non-seizure) dynamics. The individual node dynamics are governed by the stochastic complex differential equation:

(1) $$\frac{\text{dz}}{\text{dt}}=\left(a|\text{z}{|}^{4}+b|\text{z}{|}^{2}+\lambda +i\omega \right)\text{z}+\;\eta (t),$$

where ω is the parameter that controls the frequency of oscillations; λ determines the possible attractors of the system; (*a*, *b*) are real constant coefficients. The stochastic process η(t) denotes the complex noise input to the model [mean = 0.0003 and standard deviation (SD) = ±0.05], incorporating white noise to imitate state transitions driven by external or endogenous factors.

**Figure 1:**
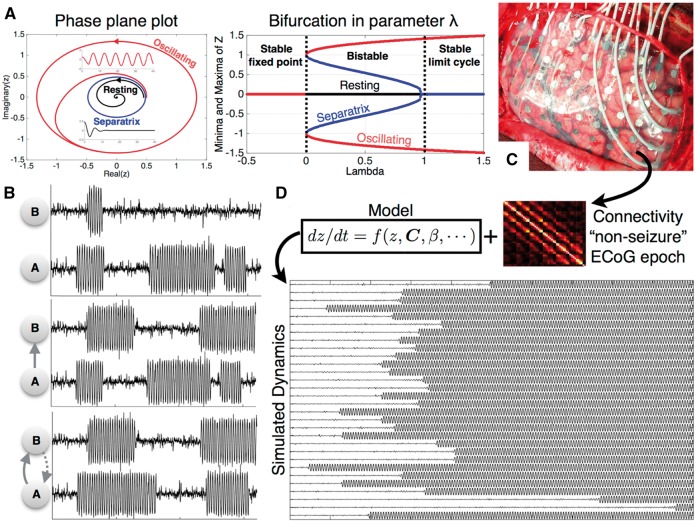
**Illustration of model dynamics.** (**A**) Deterministic dynamics of a single node representing the bi-stability of the model. (**B**) Stochastic dynamics in a two node network. The two nodes are initially disconnected having independent dynamics. Depending on the strength and direction of connections, the dynamics of each node is influenced by the other. (**C** and **D**) Patient-specific connectivity matrix is obtained from intracranial, interictal ECoG recording, which is incorporated as a model parameter to simulate the model dynamics.

Individual nodes are connected with bidirectional functional connectivity (*C*) to form a network. Therefore, the stochastic dynamics at the node level can be expanded to the network level having *N* nodes:

(2) $$\frac{{\text{dz}}_{k}}{\text{dt}}=\left(a|{\text{z}}_{k}{|}^{4}+b|{\text{z}}_{k}{|}^{2}+\lambda +i\omega \right){\text{z}}_{k}+\beta \overset{N}{\underset{j=1}{\sum }}C_{kj}{\text{z}}_{k}+\;\eta (t),$$

where, β is a scaling factor and *C* is the patient-specific functional connectivity representing functional interactions between different brain regions. Analytical treatment of this model and its implementation in the context of comparing clinical populations with controls can be found in previous studies (Kalitzin *et al.*, 2010; Benjamin *et al.*, 2012; Petkov *et al.*, 2014). In accordance with these studies, the model dynamics under different scenarios have been illustrated in Fig. 1.

It is apparent from the deterministic dynamics (without noise) in Fig. 1A that different initial conditions result in a resting (fixed-point) state or an oscillating state. The two states are separated by an unstable oscillation (sepratrix) (Fig. 1A). The model parameters are chosen such that all nodes in the model are placed in the bi-stable regime. Introducing the noise term causes the nodes to exhibit occasional transitions between the two states. This is illustrated by the two disconnected nodes A and B in Fig. 1B, whereby both nodes exhibit their independent dynamics without influencing each other. The subtleties of the model dynamics can be intuitively grasped in this simple case when the two nodes are connected to form a network. When node B is connected to node A (i.e. A→B), the dynamics of node B are influenced by A but not vice-versa. The dynamics evolve in an even more complex manner when B is also connected to A and hence influences its dynamics.

In our implementation of this model, patient-specific functional connectivity was combined with the model dynamics. The simulations exhibit transitions with focal onsets as shown in Fig. 1D. To simulate surgery, the static connectivity matrix was altered (connections to the resection site set to zero) and the model was resimulated to study the resulting change in dynamics to the remaining nodes due to the altered connectivity. This led us to make patient-specific, clinically relevant predictions which are explained in the subsequent sections. Model solutions were computed numerically using a fixed step Euler-Maruyama solver in MATLAB (The MathWorks, Natick, MA) with a step size of 0.05.

### Measure of seizure likelihood

The dynamics leading to seizure onset can be quantified using escape time of individual nodes. In our model simulations, initially all the nodes are placed in the resting state. The escape time is the time taken by a node to leave the basin of attraction in the resting state and cross over to the basin of attraction of the seizure state (Benjamin *et al.*, 2012). As the model is stochastic, the escape time τ*_i_* of each node is calculated for many different realizations of noise (i.e. η*_1_*, η*_2_* … η*_M_*). The mean escape time of each node is computed by averaging the escape time across different noise realizations. Consequently, the likelihood of a node to go into seizure is inversely related to escape time (Petkov *et al.*, 2014). In other words, a node with higher seizure likelihood has a lower escape time and therefore, also has a higher propensity to seize and vice versa. We term these simulated escape time of the network nodes presurgery as *t_prior_*.

**Figure 2:**
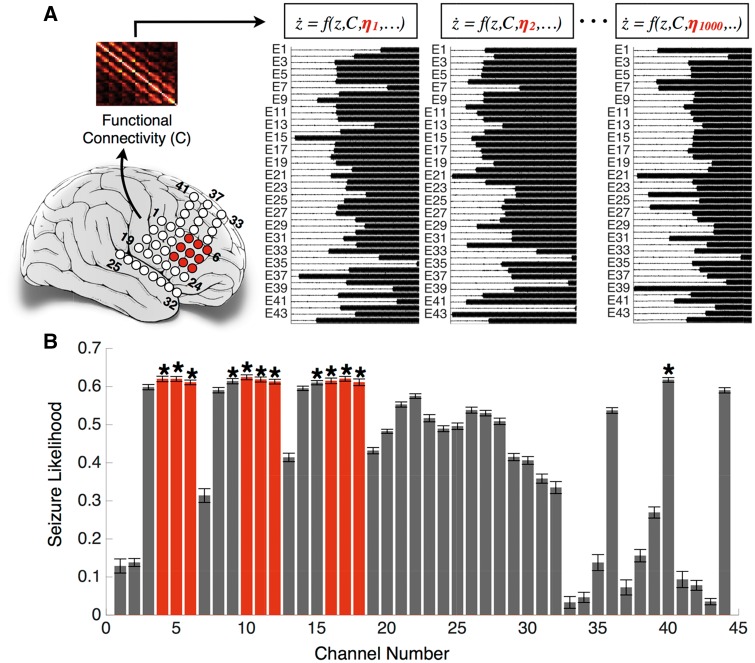
**Illustration of seizure likelihood computation.** (**A**) Electrodes in seizure onset zone (4, 5, 6, 10, 11, 12, 16, 17, 18) are shown in red on the brain schematic. The connectivity matrix inferred from the ECoG recordings is coupled with the model and the model dynamics is simulated with 1000 different noise realizations (**B**) The bar graph represents the seizure likelihood for each node and the error bars represent the standard error. Note that the nodes with significantly higher seizure likelihood (indicated by an asterisk) are correlated with the seizure onset zone shown in red in **A** and **B**.

Figure 2 illustrates the computation of escape time and seizure likelihood using the method described above. Patient-specific functional connectivity estimated from the patient’s ECoG data was incorporated as the model connectivity parameter *C* and the model was simulated with different noise realizations. Since the dynamics evolve differently for different noise realizations, the mean escape time was computed over a large number of iterations (in this case, m = 1000). Figure 2B shows the seizure likelihood for each node. Next, we delineated the set of nodes having significantly higher seizure likelihoods. We applied the non-parametric Wilcoxon rank sum test between the escape time vector of the node having the highest seizure likelihood and all other nodes. For instance, node 10 in Fig. 2 has the highest seizure likelihood, therefore, the non-parametric Wilcoxon rank sum test was applied between $\left\{\tau_{10,\;\eta_{i}}\text{|}i=1,\;2,\;\ldots m\right\}$ and $\left\{\tau_{j,\;\eta_{i}}\text{|}i=1,\;2,\;\ldots m\right\}$, where j=1, 2, …, N. The Benjamini and Hochberg false discovery rate (FDR) correction was then applied at a significance level of 5% to determine the nodes having significantly higher seizure likelihoods. These nodes are shown in Fig. 2B and were found to be correlated with the clinically determined seizure onset region denoted on the brain schematic.

### Outcome prediction criteria

Surgical intervention can be simulated in our modelling framework by altering the connectivity matrix *C*. In the model, any cortical region can be resected by setting the connectivity parameter strength to and from that region to zero. This isolates that cortical region, excluding it from contributing in the overall dynamics of the remaining network topology. The dynamical consequences of these *in silico* interventions on the remaining network can be quantified by re-simulating the model with the new connectivity matrix and comparing the changes in escape time or seizure likelihood.

We propose the following computational approach to make predictions about the efficacy of a surgical resection on seizure control and surgical outcomes. First, we need to gauge the effect of actual resection on seizure reduction in terms of model dynamics. Therefore, we alter the original connectivity matrix by removing the same network nodes as those resected clinically. With this altered connectivity we resimulate the model and note the increased values of escape time (i.e. reduction of seizure likelihood) for all the remaining nodes. We term this ‘simulated actual resection’, *t_actual_*.

Next, we posed the question: how much seizure control could have been achieved due to a resection of the same size (amount of tissue or number of network nodes) in random locations, rather than the site of actual clinical resection? To explore this, we preserved the nodes at the site of clinical resection and selected the same number of nodes randomly from the remaining set for removal from the network. The model was resimulated with this altered network and the change in escape time was noted. The same procedure was repeated and an ensemble average of the resulting escape time was taken over 100 instances to estimate the net effect on seizure reduction upon random resection. We term this ‘simulated random resection’, *t_rand_*.

Finally, we explored the ability of our method to predict surgical outcomes through the application of receiver operating characteristic (ROC) analysis and using the optimal point on the ROC curve as the threshold for classification. In order to compare the change in escape time upon removal of nodes resected clinically versus random removal of nodes, we considered the following two features: (i) difference between escape time $\left({\bar{t}}_{actual}-{\bar{t}}_{rand}\right)$; and (ii) Cohen’s *d-*score, *d_actual:rand_* to test how big the differences are in escape times. We computed Cohen’s *d*-score between two distributions *X* and *Y* as $d_{X:Y}=\;\frac{\bar{X}-\bar{Y}}{\sigma_{XY}}$, where standard deviation $\sigma_{XY}=$ mean$\left(\sigma_{X},\sigma_{Y}\right)$. The outcomes are predicted to be good if the increase in the mean escape time due to removal of clinically delineated nodes is substantially higher than that of the random resections i.e. ${\bar{t}}_{actual}>{\bar{t}}_{rand}$. Conversely, if the above condition was not satisfied, we predict that the surgery does not reduce the frequency of the seizures.

### Statistical analysis

We applied the non-parametric Wilcoxon rank sum test for comparison of escape time and seizure likelihood between the nodes. Results are declared significant for *P* < 0.05. We further applied Benjamini-Hochberg false discovery rate correction at a significance level of 5% (Benjamini and Hochberg, 1995). Cohen's *d* measures the standardized difference between two means (Cohen, 1988). Therefore, we computed Cohen’s *d*-score to measure the effect size of the variations in escape time upon resimulation of the model with altered connectivity.

## Results

**Figure 3:**
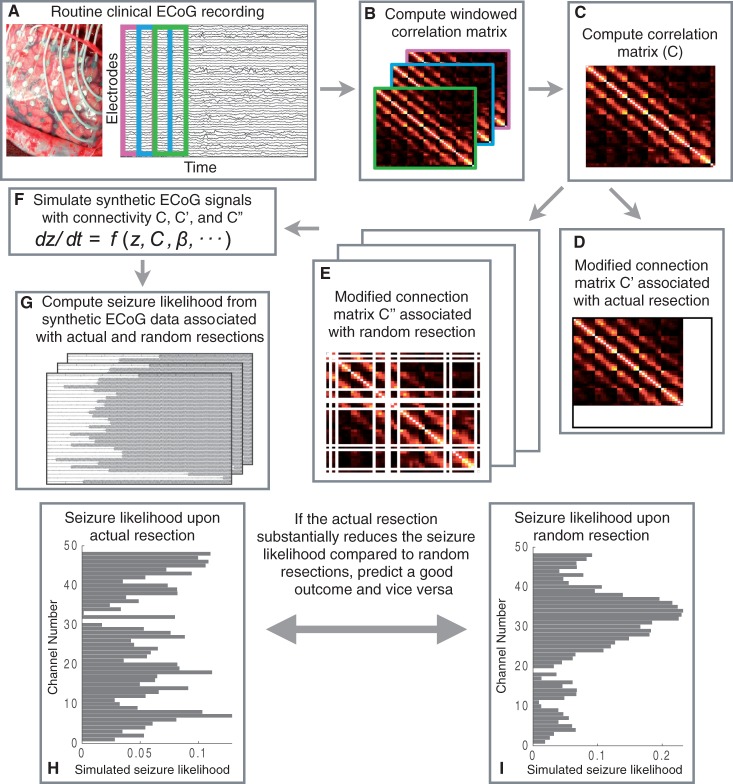
**Overall procedure.** (**A–C**) The computation of functional connectivity by averaging the windowed correlation matrices estimated from the segmented interictal ECoG signals. We coupled the model with the modified connectivity matrix from step **D** to compute the seizure likelihood upon actual resection (as shown in step **H**). Similarly, we computed seizure likelihood upon random resection (illustrated in step **I**) by coupling the modified connectivity matrix from step **E** with the model. From the steps **H** and **I**, we made predictions about surgical outcome by comparing their efficacy on seizure reduction in the model.

The results are organized into three main sections. First we attempt to identify pathological brain areas inferred from the model. Second, we reproduce the surgical procedure in the model, predict the surgical outcome, and compare the prediction to the actual outcome. Third, we predict the outcomes of alternative resections. The overall procedure is illustrated in Fig. 3. For brevity we study two representative patients; results for all 16 patients can be found in the Supplementary material.

### Pathological node identification

**Figure 4:**
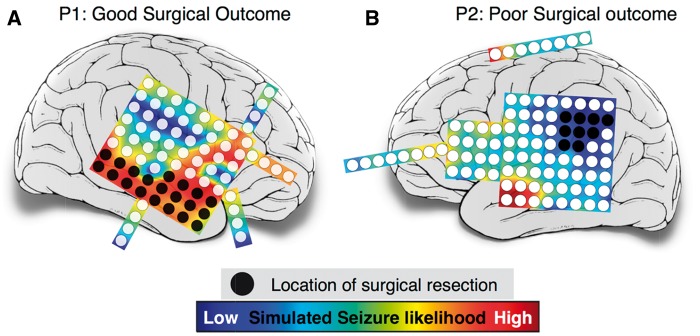
**Correlation between clinical resection, post-surgical outcome and seizure likelihood.** Cortical areas under electrode channels which were surgically resected have been shaded in black. Post-surgery, Patient P1 shown in **A** had a good surgical outcome (ILAE class II); while Patient P2 in **B** had a poor surgical outcome (ILAE class IV). The colour plot on which the electrodes are overlaid shows the distribution of simulated seizure likelihood values of different brain regions.

Clinically, the ictogenic regions of the brain are delineated mostly by visual inspection of prolonged electroencephalographic recordings. Informed by presurgical diagnostics, such as imaging and cortical mapping assessments, the tissue to be resected is circumscribed. Figure 4 shows two cases of intractable epilepsy, in which the patients were evaluated as candidates for resective surgery based on preoperative assessments. For the patient in Fig. 4A, the right temporal lobe and for patient in Fig. 4B, the left parietal cortex were diagnosed as pathological and responsible for ictogenesis. The areas clinically identified for resection are shown in black.

Surgical resection was performed clinically to remove the cortical tissues under the black shaded electrodes. The patient in Fig. 4A had improvement after surgery (ILAE class II), while the patient shown in Fig. 4B had a poor surgical outcome (ILAE class IV) with no worthwhile improvement in seizure frequency following surgery. Supplementary Fig. 1 shows seven additional cases in which the patients had good post-surgical outcomes, and seven cases in which the patients had poor outcomes after surgery. In the following we classify surgical outcome ILAE class I and II as good outcome, as both indicate a substantial and significant reduction in seizure frequency and surgical outcome ILAE class III and above as bad outcome. This way of classification is also useful when applied to the model output, as we demonstrate below.

The spatial distribution of simulated seizure likelihood in the model for different regions is coded by colour in Fig. 4. Warmer colours represent a higher propensity for seizures in the model in those brain areas. It is evident from Fig. 4A that there is a substantial overlap between the regions with high seizure likelihood and clinically delineated ictogenic regions. However, for the patient shown in Fig. 4B, our simulations predicted that the left anterior temporal cortex had higher seizure likelihood. This is in contrast to the clinically circumscribed region in the left parietal cortex. Thus, the model predictions are sometimes, but not always, in agreement with the clinically identified seizure focus. This is also the case for the other subjects studied (Supplementary Fig. 1). The result is consistent for different samples taken days apart (Supplementary Fig. 2A) and robust for specific frequency bands (Supplementary Fig. 2B). Finally, we also investigated the possible drivers behind the simulated seizure likelihood using graph-theoretic measures on the functional networks (Supplementary material and Supplementary Fig. 10). It appears that the node strength and clustering coefficient of the functional networks best explain the simulated seizure likelihood in our model.

### Prediction of surgical outcomes

**Figure 5:**
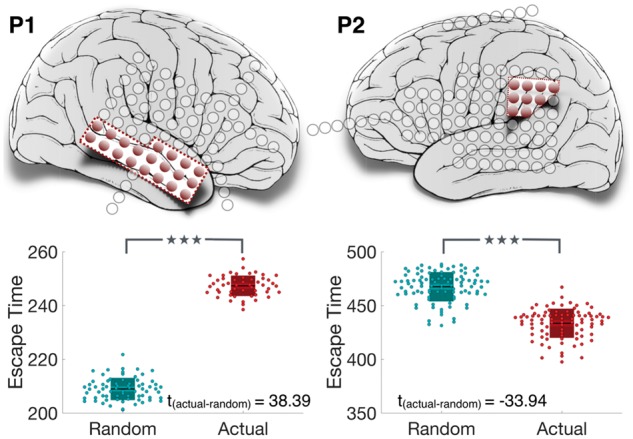
**Node removal to predict surgical outcome.** Resected cortical tissues are coloured in red. Nodes within the resected tissue are removed from the model. The resulting increase in escape time is shown in the box plot (in red), which is compared against the increase in escape time due to removal of the same number of randomly selected nodes, averaged over 100 instances (in blue). *P = 0.005–0.05; **P = 0.0005–0.005; ***P < 0.0005 computed using the non-parametric Wilcoxon rank sum test.

We proceed to demonstrate a simple yet promising computational diagnostic technique to examine the consequence of resecting a region on seizure reduction. Figure 5 shows the impact of removing brain areas on the resulting dynamics of the model for the two exemplary subjects. Due to variations between runs of the model we plot the distribution of average escape times of nodes across repeated simulations. Following the removal of brain areas in the model the escape time increases, even for randomly selected nodes. Ultimately the goal is to increase the escape time (equivalently, decrease seizure likelihood) as much as possible. The model enables us to explore the following question: does the removal of a particular set of nodes decrease the seizure likelihood more often than by chance selection of other randomly selected nodes? If so, we predict that the resection of these nodes will lead to a positive surgical outcome in the patient.

For Patient P1 (Fig. 5, left), removal of the same brain areas as those removed clinically leads to a significant increase (${\bar{t}}_{actual}>{\bar{t}}_{rand}$ and $p_{rand:actual}=1.25\;\times \;10^{-20}$) in escape time above chance removal of the same number of randomly selected nodes. Therefore, the prediction for this patient is a good outcome and agrees with empirical observations in the patient.

However, for Patient P2 (Fig. 5, right) increase in escape time due to the resection of the clinically diagnosed epileptic focus is significantly lower than chance selection of areas (${\bar{t}}_{actual}<{\bar{t}}_{rand}$ and $p_{rand:actual}=2.8\;\times \;10^{-26}$). The prediction for this patient is therefore a poor outcome and also agrees with empirical observation. Similar observations were made in other patients (Supplementary Fig. 4).

Surgical outcomes predicted retrospectively for 16 patients by applying ROC analysis is summarized in Tables 1 and 2. The ROC curves are shown in Supplementary Fig. 5 along with the area under the curve (AUC). The classification threshold was chosen such that classifier operates at the optimal point which is indicated on the ROC curve. Note that for the majority of patients (81.3%), the predicted outcome was found to be the same as the actual surgical outcome (accuracy) with 87.5% sensitivity and 75% specificity.

**Table 2:** Confusion matrix indicating performance of algorithm in predicting surgical outcomes using *t_actual:rand_*

		Actual surgical outcome	
		Seizure free = 8	Not seizure free = 8
**Predicted outcome**	**Seizure free = 9**	True positive = 7	False positive = 2 (type I error)
	**Not seizure free = 7**	False negative = 1 (type II error)	True negative = 6
	**Accuracy = 0.813**	True positive rate, or sensitivity = 0.875	False positive rate, or fall-out = 0.25
		False negative rate, or miss rate = 0.125	True negative rate, or specificity = 0.75

### Prediction for alternative resection strategies

For patients with poor predicted outcomes a key question still remains. Which areas should be removed, if any, to result in a better chance of a positive outcome?

**Figure 6:**
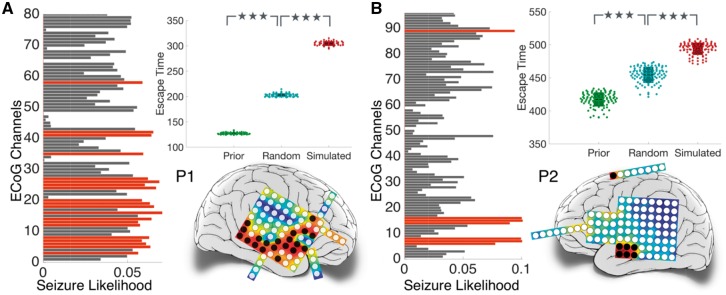
**Illustrating *in silico* approach for exploring surgical options.** The seizure likelihood for each ECoG channel is shown in the bar plot. Higher seizure likelihood indicates more propensity to seize. Nodes with significantly higher seizure likelihood after FDR correction are indicated in red in the bar plot and their spatial locations are mapped on the electrode grids in black. Nodes are removed in the model brain to simulate surgical resection. The box plots show escape time for (i) original network (in green); (ii) resection of nodes with the highest seizure likelihood (in red); and (iii) resection of same number of random nodes, averaged over 100 instances (in blue). ***P < 0.0005.

We investigated this by delineating the set of nodes having significantly higher seizure likelihood compared to the rest of the network. We refer to the ‘Measure of seizure likelihood’ section for more details on finding the nodes with highest seizure likelihood. These nodes are shown in red in the bar plots of Fig. 6. The spatial locations of these nodes on the brain schematic are indicated in black. Next, we verify whether the removal of this set of nodes minimizes seizure likelihood or maximizes escape time.

This has been demonstrated empirically in the box plots shown in Fig. 6. Note that when no nodes were removed from the model brain, the mean escape times for the patient in Fig. 6A and B were found to be ${\bar{t}}_{prior}=127.11$ and ${\bar{t}}_{prior}=417.21$, respectively. The mean escape times increased significantly (*P* < 0.0005) to ${\bar{t}}_{sim}=304.19$ and ${\bar{t}}_{sim}=493.89$ in Fig. 6A and B, respectively, upon the removal of nodes shaded in black. To determine if these set of nodes were a more favourable set delineated to minimize the overall seizures in the model, 100 sets of same order were randomly chosen. The nodes therein were removed from the model brain and in every instance the escape time was calculated. The mean escape time averaged over 100 instances is shown in Fig. 6. It is evident that for both patients, the increase in escape time due to the removal of nodes at random is significantly lower (*P* = 1.24 × 10^−20^ for Patient P1, and *P* = 4.9 × 10^−15^ for Patient P2) than the removal of nodes shaded in black (${\bar{t}}_{\text{rand}}=208.96$ for Patient P1, and ${\bar{t}}_{\text{rand}}=467.5$ for Patient P2).

We similarly investigated 16 cases (Supplementary Fig. 6) and predicted an alternative set, or subset of nodes for each patient which was corroborated with our empirical results. For each case, ${\bar{t}}_{\text{prior}}<{\bar{t}}_{rand}<{\bar{t}}_{sim}$ indicating that removal of the nodes with highest seizure likelihood would delay all the remaining nodes to transit into the seizure state, consequently reducing the overall seizure likelihood and would therefore be potentially useful for use as surgical targets. Hence, we suggest that these nodes predicted *in silico* should be considered for further investigation *in vivo* during preoperative assessments. Even in cases where our prediction overlaps substantially with the clinically diagnosed epileptic focus, our prediction often leads to a much smaller subset of these nodes, the resection of which may lead to fewer side effects.

## Discussion

In this study, we simulated the epileptogenicity of different brain regions in a mathematical model using interictal networks derived from ECoG recordings. We observe that regions with high epileptogenicity arise in the dynamical model, which often correspond to the surgically resected regions in patients who achieved seizure freedom. Indeed, we show that using the model as a predictor of surgical outcome, a sensitivity of 87.5% and a specificity of 75% (81.3% accuracy) can be achieved. In the cases where we predicted true negatives, we were further able to suggest alternative sites for resection based on the model. The methods presented here may enable clinicians to better incorporate interictal epochs of EEG in presurgical evaluation. Moreover, we have suggested a procedure for *in silico* resection, which may be helpful to locate alternative resection regions, if the seizure focus is found to be in eloquent cortex. Hence, we suggest that the epileptogenicity model can be a useful tool for measuring properties of brain connectivity networks, with easier interpretability than many traditional graph theoretic measures in the context of epilepsy.

The patient-specific functional network gives rise to the behaviour of the model and determines the epileptogenicity of each node. Consequently, the structure of this network that contains information about the underlying processes eventually produce seizures in patients. Previous literature also supports the notion that some degree of information about the epileptogenic regions are contained in these resting state functional networks (Petkov *et al.*, 2014; Schmidt *et al.*, 2014; also see van Mierlo *et al.*, 2014 for review). The mechanism underlying this phenomenon is not fully understood, although the high gamma range has been indicated to be most informative (Wilke *et al.*, 2011). A recent study highlights that structural (axonal) connectivity between regions is required for functional connections especially in the gamma band (Chu *et al.*, 2015). Taken together these studies suggest a possible structural underpinning of the functional networks found in those studies and ours.

Several earlier studies aim to predict the outcomes of epilepsy surgeries (Jehi *et al.*, 2015, see Thom *et al.*, 2010 for a review). Most existing studies focus on temporal lobe epilepsy (Schulz *et al.*, 2000; Aull-Watschinger *et al.*, 2008; Feis *et al.*, 2013; Munsell *et al.*, 2015; Coan *et al.*, 2016). A few studies reported a more heterogeneous cohort (Armon *et al.*, 1996; Asano *et al.*, 2009; Negishi *et al.*, 2011). Usually, regression analysis on routinely acquired pre- and postoperative information is performed, and significant predictors of surgical outcome are reported. Some studies additionally provide information on their prediction. Munsell *et al.* (2015) developed a predictor based on the structural connectivity of patients with temporal lobe epilepsy and report 70% accuracy. Based on MRI-derived brain morphology, Feis *et al.* (2013) present a predictor of 96% accuracy in males and 94% accuracy in females. Finally, using functional fMRI (Coan *et al.*, 2016) show a prediction sensitivity of 81% and specificity of 79% in patients with temporal lobe epilepsy. Compared to these results, our accuracy (81.3%), sensitivity (87.5%), and specificity (75%) are in a similar range. However, in case of predicting unfavourable surgical outcomes, our method was additionally able to indicate alternative areas for removal, which could result in an improved outcome. Hence, our approach goes beyond that of a simple predictor of surgical outcome, and can additionally be viewed as a complementary tool for localization in presurgical evaluation. Indeed, this is one of the key novelties of our work.

The improvement of surgical outcome has been of long standing interest in the epilepsy community. So far, it remains mostly unclear why surgery fails in some patients. Certain factors (e.g. generalized EEG abnormalities, non-lesional MRI, incomplete removal of the seizure onset zone, and secondarily generalized seizures) predispose subjects to a negative surgical outcome (Janszky *et al.*, 2000; Schulz *et al.*, 2000; Spencer *et al.*, 2005; Jeha *et al.*, 2007; Asano *et al.*, 2009; Tellez-Zenteno *et al.*, 2010). These factors are associated with a complex, possibly wide-spread epileptogenic network. Additionally, an overall more excitable surrounding cortex might also be present in some cases (Wang *et al.*, 2014), further facilitating wide-spread networks to generate seizures. This notion of excitable surrounding tissue leads to the suggestion of varying degrees of ‘healthy’, or ‘unhealthy’ tissue, rather than strict binary classifications. These conditions might lead to a more complex correlation pattern on the ECoG different from that of a classical focal seizure, which is spatially constrained and would hence show a corresponding spatially well localized correlation pattern on ECoG. This may explain the false alarm rate of 25% associated with our proposed method. In case of these complex wide-spread epileptogenic networks, additional measures of cortical excitability; e.g. using stimulus response (Valentin *et al.*, 2005) might be required to support the prediction of surgical outcome.

A further factor that might influence our results is the spatial coverage and locations of the recording electrodes. It has been reported that the number of electrodes substantially influences the inference of functional networks (Hassan *et al.*, 2014). Particularly when using ECoG (as opposed to high density EEG or MEG), parts of the brain might not be covered that are also involved in the epileptogenic network, and hence not detected. This scenario would cause our classifier to predict false positive results. A potential way around this may be to use source localization techniques in conjunction with high density EEG or MEG recordings such as the study by Englot *et al.* (2015). A so far unexplored issue is the spatial resolution of the recording. New high resolution recording modalities (Schevon *et al.*, 2008; Viventi *et al.*, 2011) might provide new insights for constructing more informative functional networks.

Traditionally, imaging information, as well as ictal ECoG, and some ECoG markers (e.g. interictal spikes) are relied upon during surgical evaluation (Rosenow and Lüders, 2001). Recently, high frequency oscillations (HFOs) have also been proposed as a marker for the epileptogenic zone to be removed at surgery (Jacobs *et al.*, 2009, 2010). Interestingly, a computational modelling study demonstrated recently that both interictal spikes and HFOs might be caused by common mechanisms, related to shifts in the balance of excitation and inhibition toward hyperexcitation (Demont-Guignard *et al.*, 2012). However, in the model, whether interictal spikes or HFOs occur depends on other factors, such as the number and spatial distribution of hyperexcitable cells. Hence, both interictal spikes and HFOs might be understood as markers of brain regions capable of occasionally generating hyperexcitable activity. As such, these areas are related, but not necessarily specific, to the seizure generating zone. In the framework of dynamic mechanisms of focal seizures (Wang *et al.*, 2014), interictal spikes and HFOs might be markers of the establishment of enabling surrounding cortex, which can support seizure activity but does not necessarily trigger or induce seizure activity. In the traditional words of presurgical evaluation, interictal spikes and HFOs would mark the irritative zone (Rosenow and Lüders, 2001), which in periods of increased excitability react with such interictal events.

In contrast, our methods here might capture a complementary signal that can be used in the presurgical evaluation. The measure of epileptogenicity we apply here is derived from minutes to hours of interictal activity. As discussed above our measure might reflect a more persistent (possibly structural) abnormality of the brain network. It is interesting to note that the modelling predictions correlate highly with predictions using the local clustering coefficient of the network nodes, and node strength (Supplementary material and Supplementary Fig. 10) suggesting localized hyperconnectivity may lead to seizure genesis in our model. Indeed, computational modelling studies have also demonstrated how local network changes can enhance or constrain spreading of activity from a focal area (Kaiser *et al.*, 2007). Hence, the significance of these model-derived and graph-theoretic measures deserve further exploration in clinical and experimental studies to fully assess its interpretation and value in presurgical evaluation.

In this study we have focused our attention on only one computational model. There is a plethora of other dynamical computational models that describe many different types of epileptic seizures (Breakspear *et al.*, 2006; Baier *et al.*, 2012; Kramer *et al.*, 2012; Taylor *et al.*, 2013; Proix *et al.*, 2014; Suffczynski *et al.*, 2004) or synchrony (Kuramoto, 1975). Indeed, the choice of model may influence the prediction outcome. In this study we simulated one of the simplest possible dynamical models of a bistability between a fixed point and a limit cycle. The choice of this model is based partly on the success of previous studies using it in conjunction with resting state electrographic data (Benjamin *et al.*, 2012) and partly because of the existence of a well-defined measure of seizure likelihood (Petkov *et al.*, 2014). Nonetheless, other mechanisms for defining seizure likelihood may be important and so future studies may benefit from the use of alternative mechanisms other than bistability. Indeed, it may be that different patients have different onset mechanisms and so multiple alternative models may capture this better (Wang *et al.*, 2014).

Our study should be considered in the context of our sample size and data. On one hand, obtaining large datasets with sufficient and accurate follow-up data is difficult and time consuming. On the other hand, obtaining multimodal, complementary imaging data (e.g. diffusion MRI, EEG, ECoG, MEG, microelectrodes etc.) is expensive. However, there have been suggestions that all of these techniques may be useful in understanding the mechanisms involved in focal seizure onset (Bonilha *et al.*, 2012; Laufs, 2012; Schevon *et al.*, 2012). While our computer model provides a framework to investigate how recordings may relate to epileptogenicity and to predict outcomes, a next step is to incorporate multimodal data for enhanced predictive value (Hutchings *et al.*, 2015). This requires extensive data collection, annotation and analysis. We have demonstrated the potential of this approach and suggest that a larger study of model-based prediction of surgical intervention may prove useful.

A key benefit of our approach is to predict alternative resection strategies. To our knowledge our use of a dynamical model based approach to do this is entirely novel. Indeed, this would be highly beneficial not only when the prediction is of a poor outcome for the suggested site, but also when the suggested site is located in eloquent cortex (e.g. motor/language areas). Another benefit of our approach is that we incorporate interictal segments of routinely collected clinical data. Usually patients are implanted in order for the clinician to observe seizures on the electrodes to decide which areas the seizure originates from. This can lead to long hospitalization times since it can be highly unpredictable when the seizures will occur. The use of interictal data is therefore potentially beneficial and complementary to imaging through the use of ictal data and MRI. The incorporation of our patient-specific model output into the clinical decision making process, in the future, may lead to improved surgical success and mechanistic insight into the pathophysiology of seizures.

## Supplementary Material

Supplementary Data

## Abbreviation

ECoG: electrocorticography
