# Supplementary material for: Predicting neurosurgical outcomes in focal epilepsy patients using computational modelling
Source: Brain. 2016 Dec 30;140(2):319–32. doi: 10.1093/brain/aww299 (PMC5278304; doi:10.1093/brain/aww299)
Supplement: Supplementary Data [file aww299_supp.pdf]

Supplementary Figure S1: Location of surgical resection, post-surgical, outcome and simulated seizure likelihood.

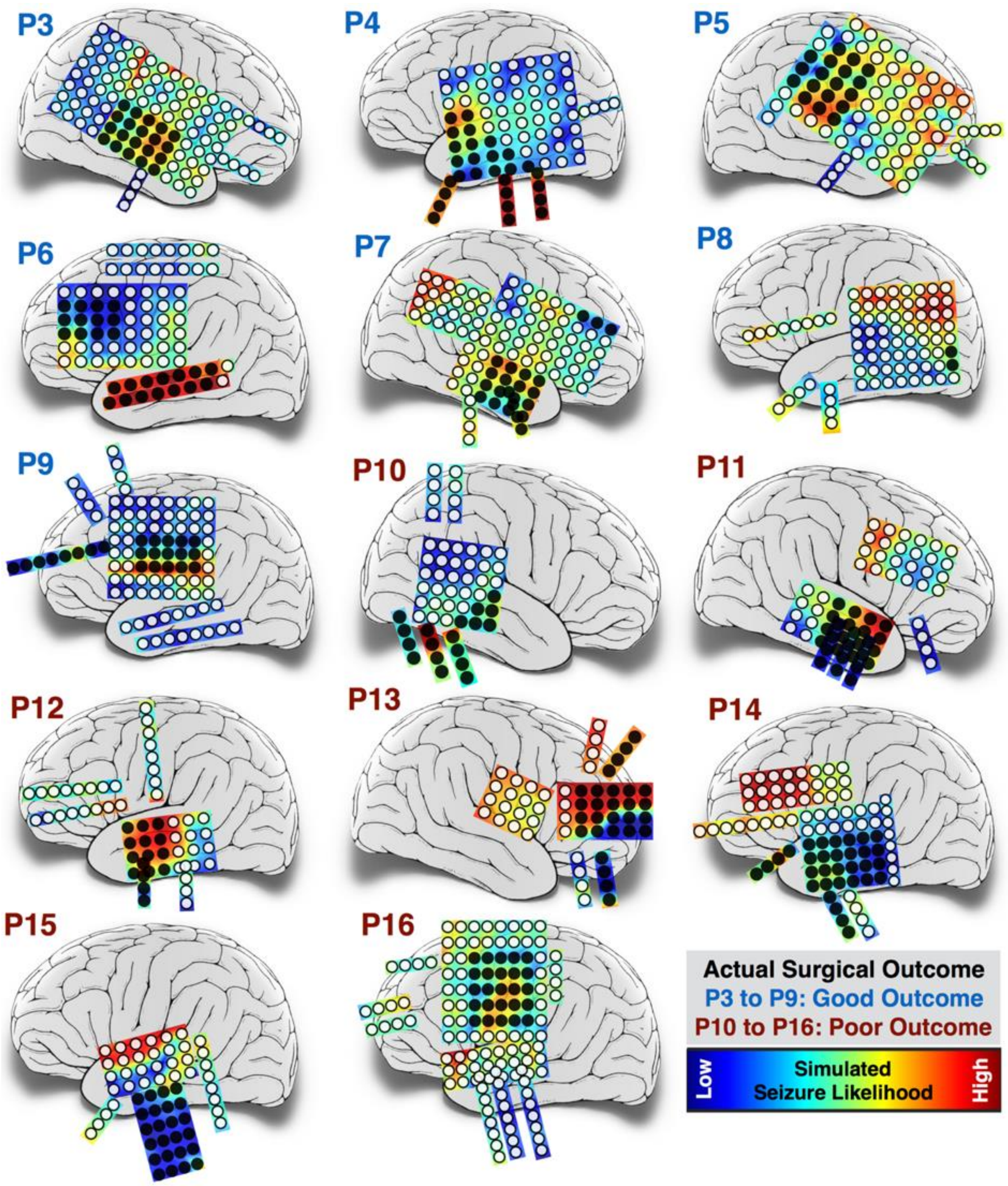

Supplementary Figure S1: Location of surgical resection, post-surgical outcome, and simulated seizure likelihood. Further to the two exemplary cases shown in Fig. 4, fourteen more cases are shown here. P3 to P9 correspond to cases in which patients were rendered

seizure free, while for cases P10 to P16, seizures persisted post-surgery. Electrodes in black indicate the location of surgical resection, whereas the color plot represents computed seizure likelihood for different cortical regions.

Supplementary Figure S2: Consistency across different inter-ictal segment and frequency bands.

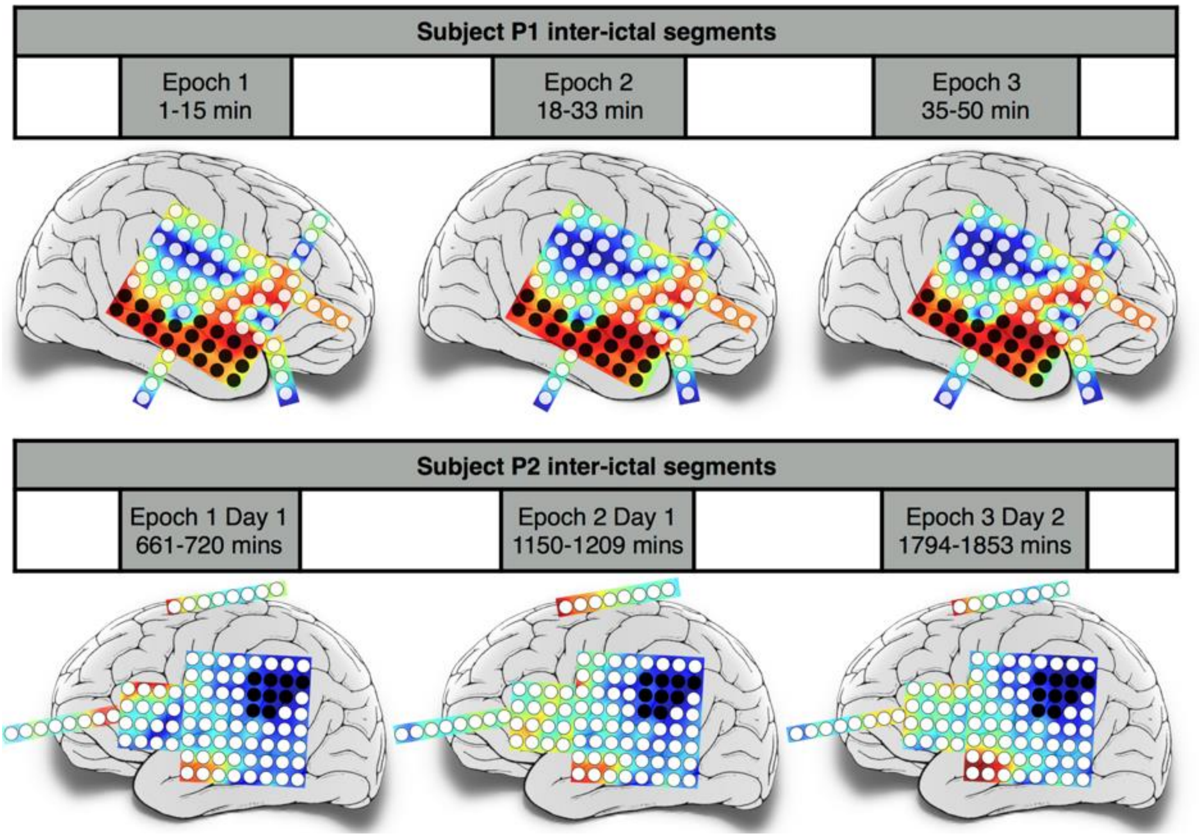

Figure S2a: Different inter-ictal segments

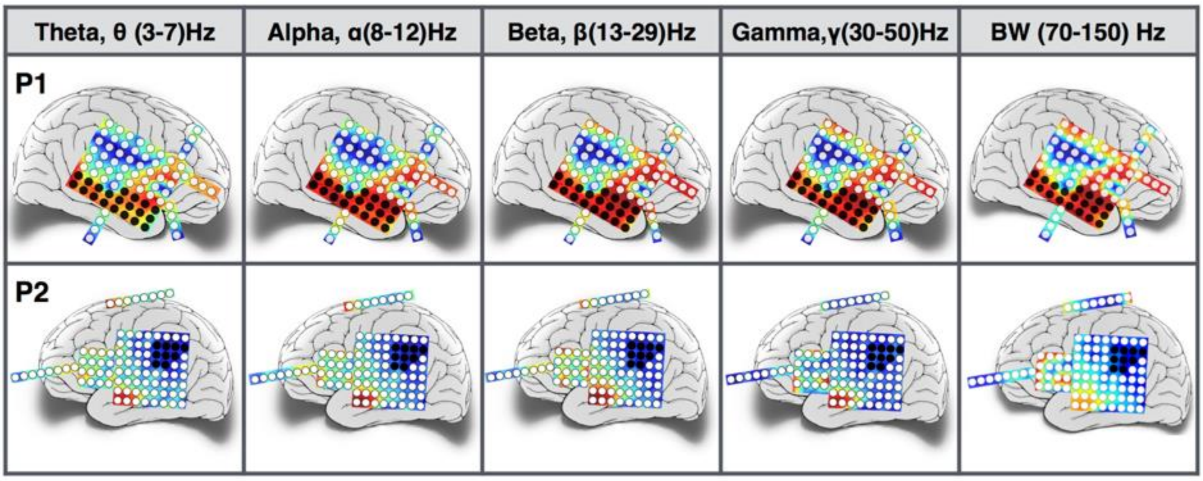

Figure S2b: Different frequency bands

Supplementary Figure S2: Consistency across different inter-ictal segments and frequency bands. The upper panel depicts the distribution of seizure likelihood for different inter-ictal

segments. For patient P1 the availability of inter-ictal data was constrained to one hour. Consequently, we limited the epoch segments to 15min duration. Patient P2 underwent two days of intracranial investigation; the first 1-hour epoch segment has been selected from the beginning of monitoring and the other two epochs are chosen from different days. The lower panel illustrates the distribution of seizure likelihood for $\theta, \alpha, \beta, \gamma$ frequency bands and a frequency band from 70-150 Hz. The escape time depends on the frequency band but the distribution of seizure likelihood remains unaffected. Therefore, it is not necessary to choose specific frequency bands in order to make predictions.

Supplementary Figure S3: Raw ECoG signals from randomly chosen electrodes.

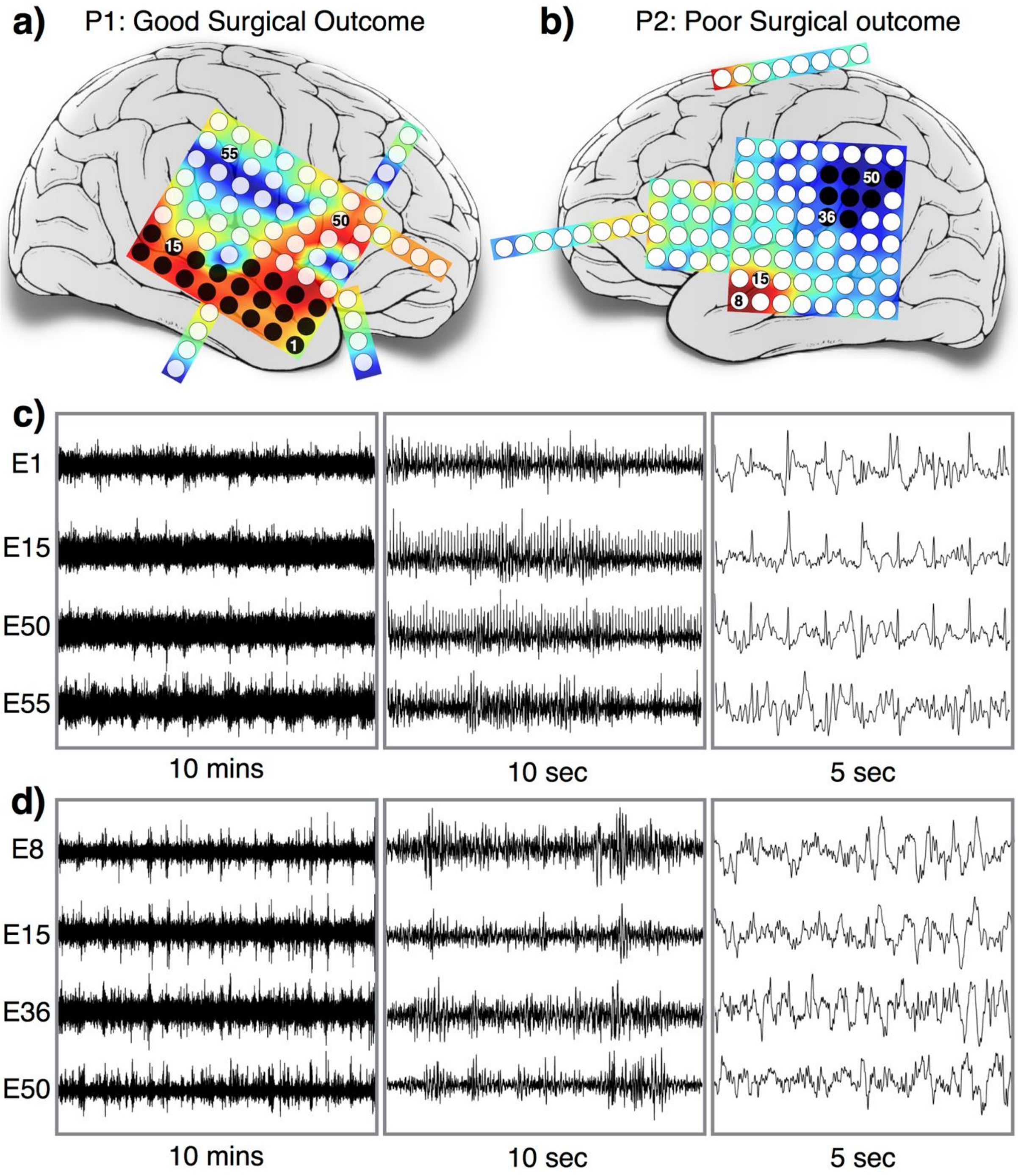

Supplementary Figure S3: Raw ECoG signals from randomly chosen electrodes. Panel (a) shows four randomly chosen electrodes from the ECoG grid of patient P1 and P2. The ECoG signals are shown at a resolution of 10min, 10s and 5s. It is apparent that there is no unusual difference between the raw inter-ictal signals from the electrodes placed at

different locations on the cortex.

Supplementary Figure S4: Prediction of surgical outcomes by simulating resections.

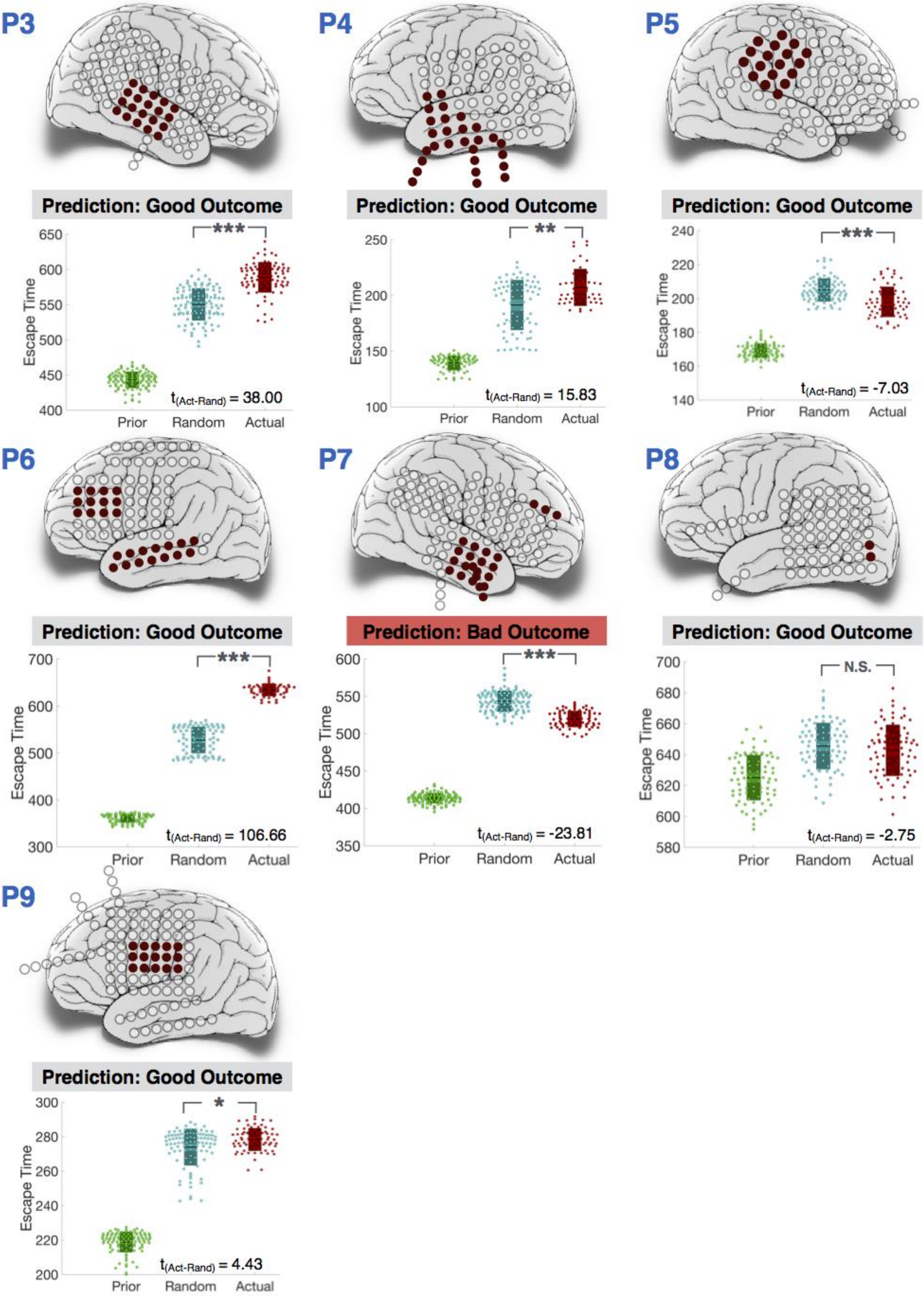

Figure S4(a): Subject P3 to P9

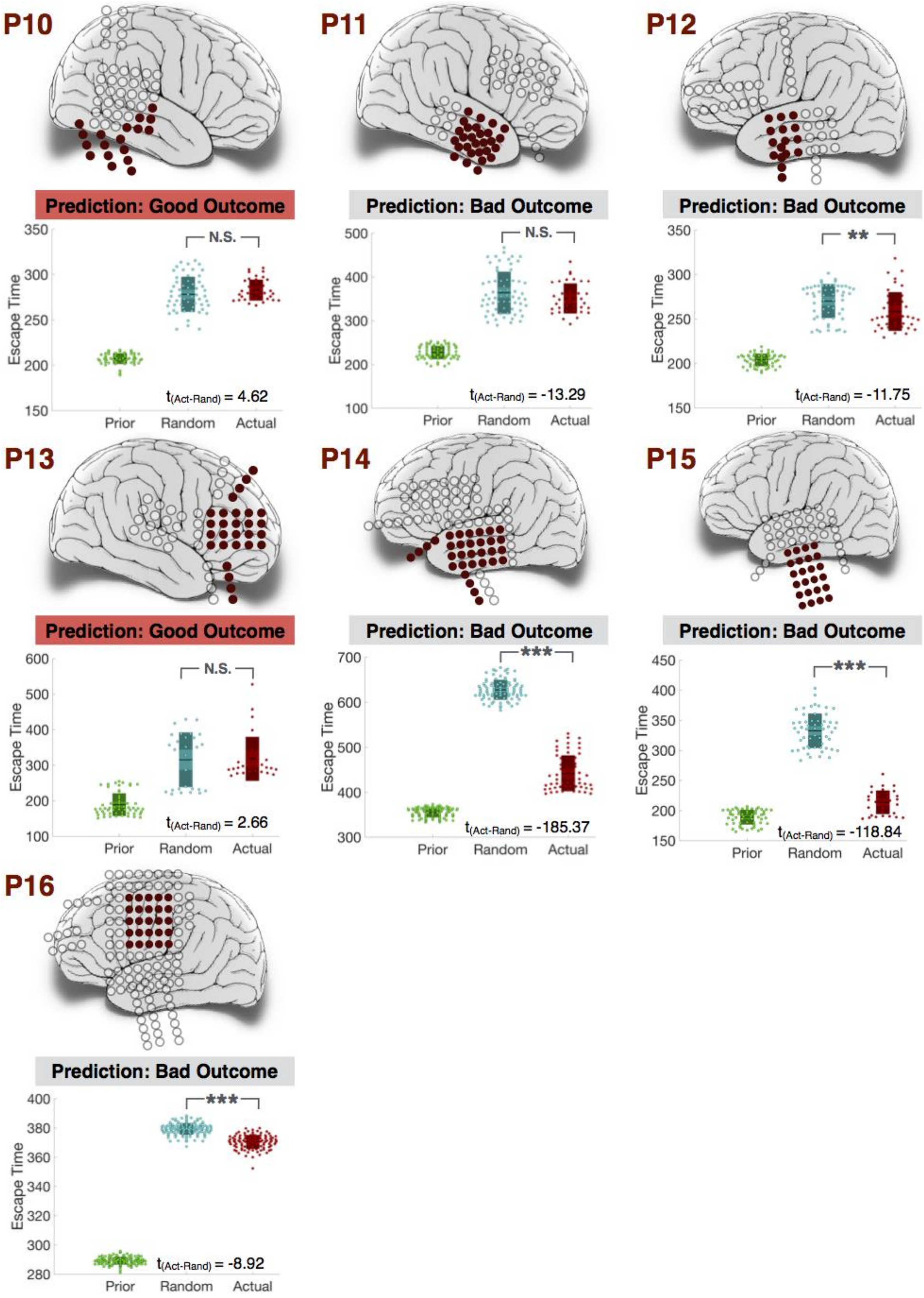

Figure S4(b): Subject P10 to P16

Supplementary Figure S4: Prediction of surgical outcomes by simulating resections. This

figure illustrates the prediction of surgical outcomes for fourteen more patients; it is the equivalent of Fig. 5 where the same is shown for two exemplary cases. In each case, we have removed the nodes within the resected cortical tissues from the model. We compared the consequent increase in escape time with that of the random node removals and made predictions which are mentioned corresponding to each patient.

Supplementary Figure S5: Receiver operator characteristic analysis

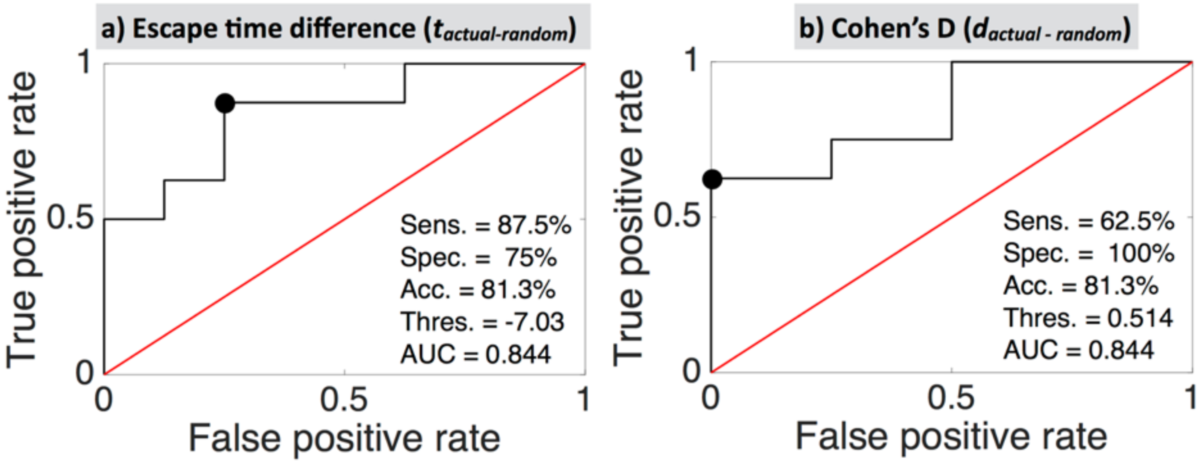

Supplementary Figure S5: Receiver operator characteristic analysis. Receiver operating characteristic for the prediction of good and bad surgical outcomes using difference in escape time and *d-score* as a classification feature. The threshold value for optimal classification is indicated in both the figures and the black dot denotes the point with best classification which is the point closest to (true positive rate = 1, false positive rate = 0). Abbreviation: Sens.– sensitivity, Spec.– specificity, Acc.– accuracy, AUC– area under the curve, Thres.– threshold for classification.

Supplementary Figure S6: Exploration of alternative resection strategies.

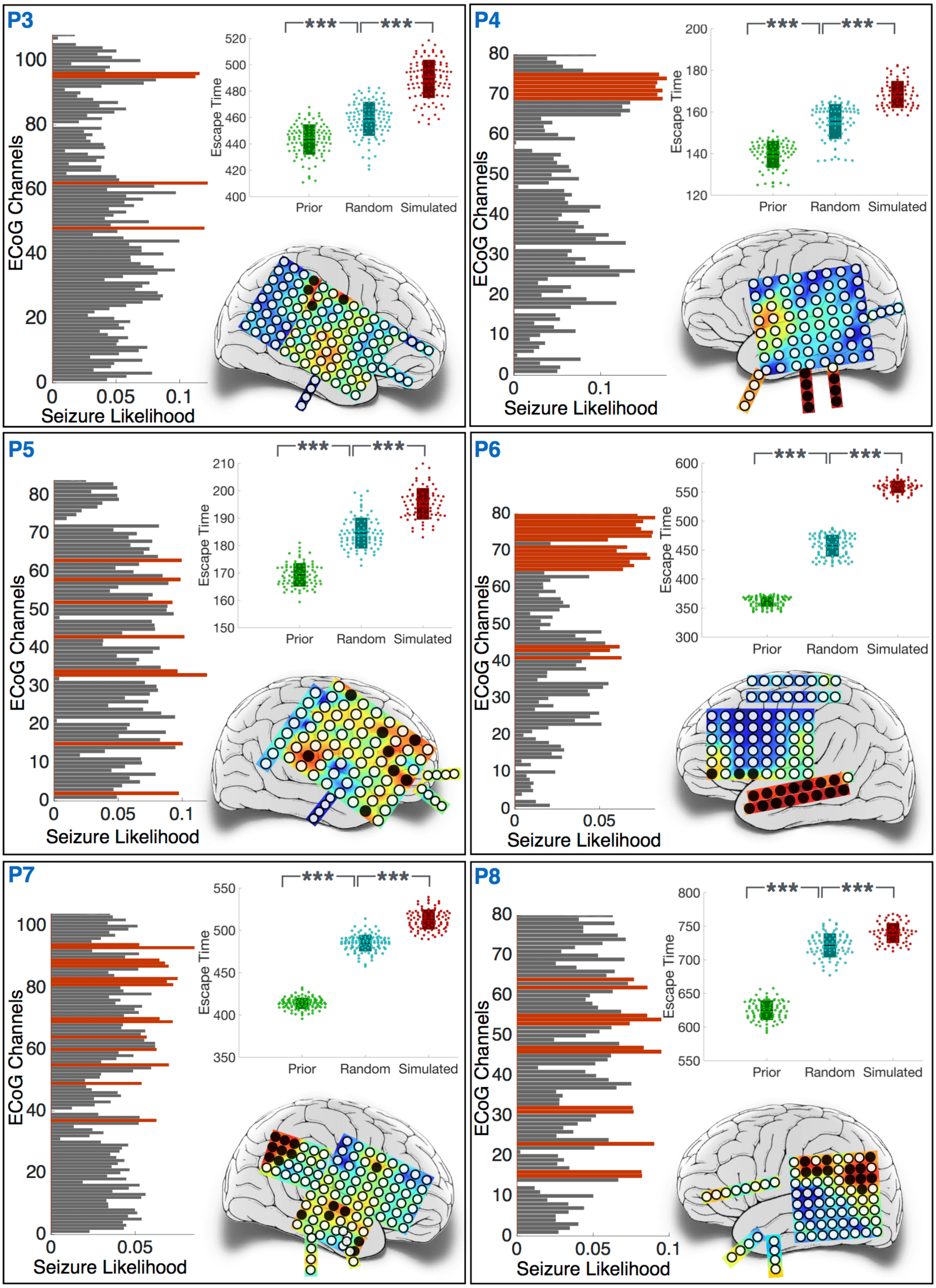

Figure S6(a): Subject P3 to P8

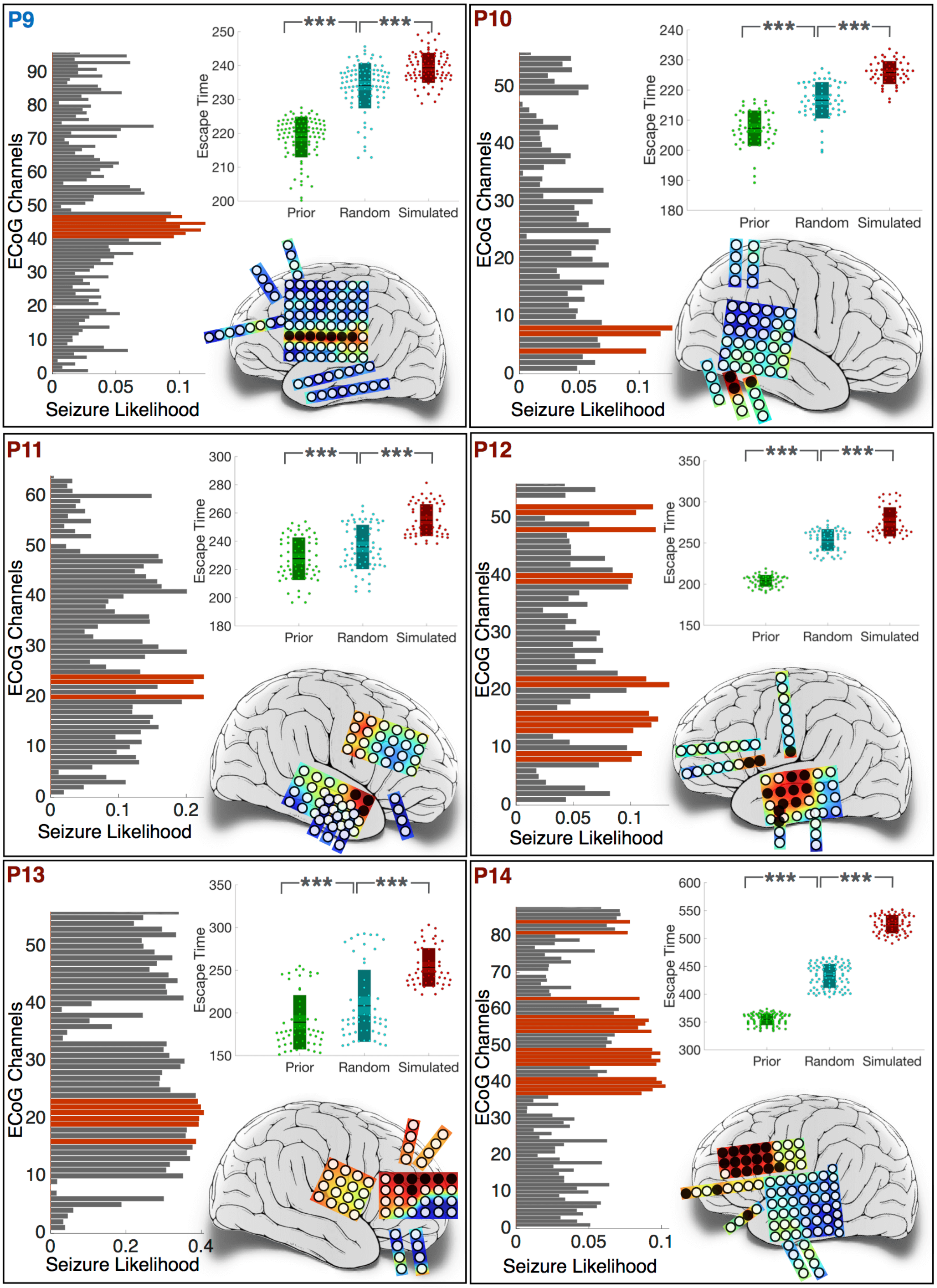

Figure S6(b): Subject P9 to P14

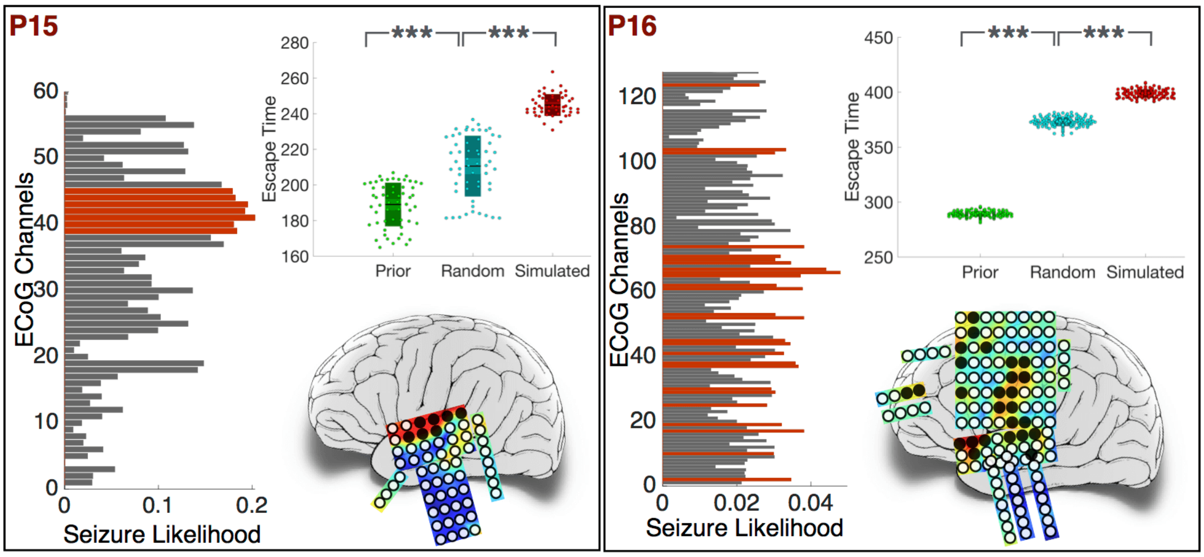

Figure S6(c): Subject P15 to P16

Supplementary Figure S6: Exploration of alternative resection strategies. This figure depicts the *in silico* approach we propose for the exploration of different surgical options. It is the equivalent of Fig. 6 for fourteen additional cases. For each case, we compute the set of nodes with highest seizure likelihood which are shaded in black. The box plot represents the increase in escape time upon removal of these nodes compared against the removal of random nodes from the model.

Supplementary Text S7: Additional information about the patients provided by the IEEG portal.

The details of following patients used in this study have been adapted from the IEEG portal. Further details on these patients can be found at https://www.ieeg.org

**Subject P2**	
IEEG ID	Study 028 (by Mayo Clinic)
Inter-ictal Segment	1794 – 1853 min
Seizure Events	3 typical seizures in 2 days of intracranial monitoring
Seizure Description (onset and spread)	Epileptiform Discharges: Arising from the left parietal grid at contact LPG 52 with field spread to contacts LPG 51, 53, 59, 60, and 61.<br>Seizure onset and spread: LPG 44, 51, 52, 53, 59, 60, 61
Resection Description	Left Parietal Cortex, 11g (3.3 x 3.2 x 1.8) cm
Surgery Commentary	We outlined our proposed cortical resection from the superior aspect of the grid centred on electrode 52 but extending down to more leads for 2 cm and then posteriorly 4 cm in a triangular-shaped fashion. We made efforts to protect the central sulcus vein. Then once we had marked the outline of the cortical resection boundaries with silk suture, this boundary was bipolar cauterized down to the pia. Then using microsurgical technique, we performed a corticectomy and deepened it to the white matter circumferentially around the proposed boundary. Once we had circumferentiated the entire area, taking extra care to protect the large central sulcus vein, we undercut the cortical resection through the white matter using bipolar cautery, taking care not to enter the ventricle and staying deep to the grey matter within the central sulcus.
Location of Resection	LPG 35, 36, 42, 43, 44, 49, 50, 51, 52

**Subject P6**	
IEEG ID	Study 038 (by Mayo Clinic)
Inter-ictal Segment	1471 – 1530 min
Seizure Events	3 typical and 6 subclinical seizures in 4 days of intracranial monitoring
Seizure Description (onset and spread)	2 typical seizures Left Frontal onset, 1 typical seizure Left Temporal Neocortical onset, 6 subclinical Left Mesial Temporal Depth onset.<br>Epileptiform discharges: LG 1-16, 17-20, 27 and 35, IIS 3-6, SIS 3-6, Frequent but not continuous STS 1-5, Occasionally in AD, PD<br>Seizure 1: Typical; ITS 1-4, STS 3-7<br>Seizure 2: Subclinical; AD 1, Spreads to AD 2-4, PD 3, 4

	Seizure 3: Subclinical; AD 1, Spreads to AD 2-4, PD 3, 4<br>Seizure 4: Subclinical; AD 1, Spreads to AD 2-4, PD 3, 4<br>Seizure 5: Subclinical; AD 4, Spreads to AD 1-3, PD 3, 4<br>Seizure 6: Typical; LG 9-12, 17-20, 25-28, Spreads LG 1-4, ITS 1-8, STS 1-8<br>Seizure 7: Subclinical; AD 1, Spreads to AD 2-4, PD 3, 4<br>Seizure 8: Typical; LG 9-12, 17-20, 25-28, Spreads LG 1-4, ITS 1-8, STS 1-8<br>Seizure 9: Subclinical; AD 4, Spreads to AD 1-3, PD 3, 4
Resection Description	Left Hippocampus: 1.52g, (2.4 x 1.2 x 1) cm<br>Left Medial Frontal Lobe (partial resection): 11.5g, (4.6 x 2.7 x 1.6) cm
Surgery Commentary	We first turned our attention to the middle fossa. We identified the temporal horn of the lateral ventricle and carried out an amygdalohippocampectomy. The resection edges were lined with Surgicel. We then turned our attention to the frontal region. A cortical resection was outlined of the epileptogenic onset zone and extended to the falx and anterior to the frontal pole. We did excise a region of frontal encephalomalacia adjacent to the epileptogenic onset zone.
Location of Resection	LG 9-12, 17-20, 25-28, STS 1-7, ITS 1-7

**Subject P7**	
IEEG ID	Study 021 (by Mayo Clinic)
Inter-ictal Segment	4901 – 4960 min
Seizure Events	9 clinical and 2 subclinical seizures in 4 days of intracranial monitoring
Seizure Description (onset and spread)	Onset from right frontotemporal neocortex, 6 seizures from diffuse right frontotemporal onset, 5 seizures from right frontotemporal, right temporal, and right frontal onset.<br>Epileptiform discharges: RFG 28-32, 35, 37-41, 44-48, 1, 2, 9-12, and 14, RFG 1, 9, 10, 41, 28, 29, RTG 19 and 20.<br>Seizure 1: Typical; RFG 41-43, RAT 2-3, RTG 1-4, 7-12<br>Seizure 2: Typical; RFG 41,43, 44, RAT 2, RTD 1, Spreads to Right Temporal Grid<br>Seizure 3: Subclinical; RFG 46-48, Spreads RAT 2, RTD 1-4<br>Seizure 4: Typical; RFG 30-32, 46-48, RAT 2-4, RPT 1-3, Spreads to RTG<br>Seizure 5: Typical; RFG 30-32, 46-48, RAT 2-4, RPT 1-3, Spreads to RTG<br>Seizure 6: Typical; RTG 2-4,10, 22, 23 RFG 31-32, RAT 1-4, RPT 1-4, RTD 1<br>Seizure 7: Typical; RFG 46-48, RAT 1-4, RTD 1-4<br>Seizure 8: Typical; RAT 2<br>Seizure 9: Typical; RAT 2<br>Seizure 10: Subclinical; RFG 46-48, Spreads RAT 2, RTD 1-4<br>Seizure 11: Typical; RFG 44-48, RAT 2, RTD 1, Spreads to Right Temporal Grid

Resection Description	Right temporal lobectomy<br>Right amygdalohippocampectomy
Surgery Commentary	N.A.
Location of Resection	RTG 1-4, 7-10, 13-16, 19-22, RAT 1-4

**Subject P8**	
IEEG ID	Study 023 (by Mayo Clinic)
Inter-ictal Segment	481 – 540 min
Seizure Events	4 complex partial seizure in 2 days of intracranial monitoring
Seizure Description (onset and spread)	Onset from left posterior occipital grid<br>Epileptiform discharges: LTG 1, 3, 4, 21, 22, 23, 25-28, 30-31, 38-40, 50-52, 57-60, 63 spreads to ITS 2, AD 1,2, PD 1,2.<br>Seizure onset: LTG 59, 58, Early spread: LTG 2, 3, 10, 11, 18, 19 Late Spread: LTG 50, ATS 3,4 AD 1,2, PD 1-4.
Resection Description	Left occipital brain lobe: 7.51g, (5.2 x 2.8 x 0.8) cm
Surgery Commentary	An 11 blade was taken, and attention was first paid to the cortical region corresponding to numbers 58 and 59 on the large 8 x 8 subdural grid. The cortex was sharply incised, and dissection was performed using bipolar cautery and microscissors. Dissection proceeded circumferentially to include the left occipital pole. It was taken down until the falx came into view. At this point, vigorous bleeding from venous lakes were encountered.
Location of Resection	LTG 58, 59

**Subject P9**	
IEEG ID	Study 026 (by Mayo Clinic)
Inter-ictal Segment	365 – 424 min
Seizure Events	N. A.
Seizure Description	N. A.
Resection Description	Left lateral frontal cortex: 16g (3.5 x 3.5 x 1.9) cm<br>Left anterior frontal cortex: 3g (2 x 1.7 x 1.6) cm<br>Left medial frontal cortex: 2.09g (2.6 x 1.5 x 1.1) cm
Surgery Commentary	Using the electrodes as guides, we then mapped out the area that the seizure focus mapped to, and the grids were subsequently removed from the brain. Using a combination of bipolar cautery and suction, the neocortex was removed. This was taken down to the white matter in a posterior-to-anterior fashion until all the neocortex was removed. Next, we turned our attention to the interhemispheric region and deepened this resection cavity all the way down to the corpus callosum

	again using bipolar cautery and suction.
Location of Resection	LFG 33-40, 41-48, LFP 1-8

**Subject P10**	
IEEG ID	Study 004-2 (by Mayo Clinic)
Inter-ictal Segment	9429 – 9488 min
Seizure Events	N.A.
Seizure Description	N.A.
Resection Description	Right Temporo-Occipital 5.29g (4.2 x 2.2 x 1.3) cm
Surgery Commentary	The parietal strip electrodes in the grid were removed leaving only the subtemporal strips in place. We then inspected the cortex underlying the active electrode contacts and ultimately fashioned a cortical resection which was contiguous with the previous posterior temporal resection extended subtemporally to include the entire area of contact with the strip electrodes. Posteriorly, the active electrode sites off of the posterior strip abutted a moderate sized vein draining the occipital lobe. This vein was left intact and functioned as a posterior boundary for the resection. We did enter the atrium of the ventricle.
Location of Resection	RAT 1-4, RMT 1-4, RPT 1-4, RG 1-3, 7, 8, 13

**Subject P11**	
IEEG ID	Study 016 (by Mayo Clinic)
Inter-ictal Segment	2601 – 2660 min
Seizure Events	5 typical and 2 sub-clinically seizures in 2 days of intracranial monitoring
Seizure Description<br>(onset and spread)	Onset at right temporal regions and right orbito-frontal regions<br>Epileptiform discharges: Seizures like activity were most prominent in RTG 4-22, RPS, RAS, RMS and ROS<br>Seizure 1: RTG# 22, 23; Spreads to RTG# 9-16, ROF# 1-4<br>Seizure 2: RTG# 7; Spreads to ROF# 3<br>Seizure 3: RTG# 1, 5, 9; Spreads to RAS# 1, 2<br>Seizure 4: ROF# 3, Spreads to RAS# 1-4<br>Seizure 5: Onset not clear but may be in ROF# 1-4, Spreads to RTG# 6<br>Seizure 6: ROF# 1-4<br>Seizure 7: ROF #2-4
Resection Description	Right Temporal Lobectomy (extended) 65 mm
Surgery Commentary	Extended right temporal lobectomy to what appeared to be the junction between the temporoparietal and occipital lobes. There was no vein of Labbe. However, there was a large posterior temporo-occipital branch which served functionally as

	the posterior margin of the resection. At the completion of the resection, the total lobectomy now measured 65 mm.
Location of Resection	RTG 9 – 24, RPS 1-4, RMS 1-4, RAS 1-4

**Subject P12**	
IEEG ID	Study 029 (by Mayo Clinic)
Inter-ictal Segment	2001 – 29060 min
Seizure Events	3 typical secondarily generalized seizures in 6 days intracranial monitoring
Seizure Description (onset and spread)	Onset at Left Mesial Temporal lobe<br>Epileptiform discharges: Right temporal depth electrode and subtemporal mesial contacts<br>Seizure 1: AIT 1-4, PIT 1-4; Spreads to LT 1-24<br>Seizure 2: AIT 1-4, PIT 1-4; Spreads to LT 1-24<br>Seizure 3: AIT 1-4, PIT 1-4; Spreads to LT 1-24
Resection Description	Left anterior temporal lobectomy 13.5g (4.2 x 3.4 x 1.7) cm<br>Amygdalohippocampectomy 1.2g (1.7 x 1.5 x 1) cm
Surgery Commentary	We then removed all the electrodes and washed the subdural space again with antibiotic solution. We brought the operating microscope into the field, and under the scope using microtechnique, a left anterior temporal lobectomy was carried out to 30 mm from the temporal tip. The amygdala and hippocampus were removed as separate specimens.
Location of Resection	LT 1-3, 7-9, 13-15, 19-21, AIT 1-4

**Subject P13**	
IEEG ID	Study 020 (by Mayo Clinic)
Inter-ictal Segment	4565 – 4626 min
Seizure Events	4 typical and 5 sub-clinically seizures in 5 days of intracranial monitoring
Seizure Description (onset and spread)	Right Anterior Frontal Onset<br>Epileptiform discharges: Polyspikes were seen at electrodes RAG 2, 3, 8, 9. SWD occurred most frequently at electrodes RAG 8, 9, 10 and were also seen at contacts RAG 2, 3, 4, 5, 7, 11, 13, 14, 15, 16, 17, RAF 2, 3, 4, RPF 3, 4, 14, RPG 9, 10, 11, and RAI 2. Low amplitude fast activity occurred frequently in electrodes RAG 1, 2, 3, 7, 8, 9, 10, 11, 13, 14, 15, 17, RAF 2, 3, and RPF3.<br>Seizure 1: Typical; RAG 8, 9, 10, 15, 16, 17 spreads to RAG 14, 20, 21, 22, 23, 24<br>Seizure 2: Typical; RAG 5, 7, 8, 9, 10, 14, 15 spreads to all channels in RAG<br>Seizure 3: Typical; RAG 7, 8, 9, 10, 11<br>Seizure 4: Typical; RAG 2-5, 8-10, 13-18 Spreads to all channels in RAG

	Seizure 5-9: Subclincal had similar onset.
Resection Description	Right Frontal lobe: 15.94g (5.3 x 3.6 x 1.1) cm
Surgery Commentary	The primary areas of interest included nos. 8, 9, and 10, as well as 5 and 11, on this 4 x 6 grid. There are also areas of clinical spread on the anterior inferior frontal strips, as well as the anterior superior frontal strips. It was felt that all of these areas of clinical spread, as well as the initial seizure focus, could be safely removed with an anterior right frontal lobectomy. We compared the venous anatomy to the previously placed grids. We identified a right anterior frontal vein that coursed through electrode 24 on the 4 x 6 grid. We bipolared the cortex just anterior to this vein all the way from the superior margin down inferiorly to near electrode 6 on the 4 x 6 grid. This margin included all the aforementioned foci for seizure activity. We then proceeded to corticectomy at this margin.
Location of Resection	RAG 1-5, 7-11, 13-17, 19-23, RAF 1-4, RAI 1-4

**Subject P14**	
IEEG ID	Study 019 (by Mayo Clinic)
Inter-ictal Segment	5261 – 5320 min
Seizure Events	41 seizures (13 complex partial seizures, 3 secondary generalised seizures, 25 subclinical seizures) in 6 days of intracranial monitoring
Seizure Description (onset and spread)	Multiple independent left temporal foci<br>Spike wave Discharges: LT 1, 2, 7, 8, 16, 17, 22, 23, 33, 34.<br>Seizure 1-8, 18-22: LT 15, 16; PT 4<br>Seizure 9-17, 33, 34: LT 1, 2<br>Seizure 23, 24: LT 2, 3<br>Seizure 25-28, 30-32, 35, 36, 38, 40, 41: LT 21, 22<br>Seizure 29, 37: LT 1-7<br>Seizure 39: AT 1, 2
Resection Description	Left Temporal neocortical resection with length approximately 7 cm
Surgery Commentary	We were concerned about intimacy of epileptic zone with speech. Significant risk of speech dysfunction. We performed a modified resection of the lesser trochanter left temporal neocortex. We removed brain tissue both anterior to and posterior to the vein of Labbe. The vein Labbe was protected and preserved in continuity with the bridging bit of brain. We did undermine that bridging bit of brain. The amygdala and hippocampus were not removed.
Location of Resection	LT 1-4, 7-10, 13-16, 19-22, 25-28; AIT 1-4; AT 1-4

**Subject P15**

IEEG ID	Study 022 (by Mayo Clinic)
Inter-ictal Segment	426 – 485 min
Seizure Events	7 complex partial seizures in 4 days of monitoring
Seizure Description (onset and spread)	Anterior temporal and inferior temporal onset<br>Spike wave discharges over ATS 1-4, PTS 6-8. Frequent discharges over TIG 1-3, 7-9, 3-15 and TSG 11,12,14,19-21,22-24.<br>Seizure 1: ATS 1-4, TIG 1-3, 5, 6, 7-10, 13-17, 21-24<br>Seizure 2: ATS 1-4, TIG 1-3, 7-10, 13-16 spreads to PTS 1-8, TSG 21-23, TIG 1-24<br>Seizure 3: ATS 1-4, TIG 1-3, 7-10, 13-16 spreads to TIG 5, 6, 17, 18, 23, 24<br>Seizure 4: ATS 1-4, TIG 1-3, 7-9, 13,19 spreads to TIG 4-6, 10-11, 20-22, PTS 3-8<br>Seizure 5: ATS 1-2, 4 TIG 1-3, 6, 7, 8, 13-15, 17, 18, 23, 24; spreads to 4, 5, 9-12<br>Seizure 6: ATS 2-4 TIG 4-6, 11, 12, 23, 24<br>Seizure 7: TIG 1-3, 7, 8 ATS 2-4
Resection Description	Left Temporal Lobe: 10.8g (5.2 x 3.5 x 0.6) cm
Surgery Commentary	All of the left temporal neocortex from the middle, inferior, and fusiform gyri down to the collateral sulcus was removed from the temporal tip back to the vein of Labbe. Therefore, this would be considered a modified neocortical left temporal lobectomy.
Location of Resection	TIG 1-24

**Subject P16**	
IEEG ID	Study 033 (by Mayo Clinic)
Inter-ictal Segment	8301 – 8360 min
Seizure Events	Innumerable complex partial and subclinical seizures in 7 days of monitoring
Seizure Description (onset and spread)	Central portion of Left Frontal Grid<br>All Seizure starts from LG 19-23, 27-31, 35-39, 43-47, 51-55 and spreads to LG 9-10
Resection Description	Left Frontal Lesion, 29.9g (5 x 4.6 x 2.2) cm
Surgery Commentary	Identified an area from approximately 51-55, 43-47, 35-39, 27-31,19-23 where the underlying abnormality was noted. We then used those grids to identify sulcal and gyral pattern consistent with this area of seizure onset zone. We outlined the area using bipolar cautery, and then the grid was removed. We deepened the resection around the margins of this area with bipolar cautery, maintaining excellent hemostasis, and were eventually able to remove a large area of the left frontal lobe. Portions of it, particularly that were superior and posterior in this region, were particularly hard, most consistent with the underlying tuber, and this was confirmed with the stereotactic registration.

Location of Resection	LG 19-23, 27-31, 35-39, 43-47, 51-55

Supplementary Text S8: Applying Laplacian and Bipolar montage for ECoG pre-processing.

1. Laplacian Montage

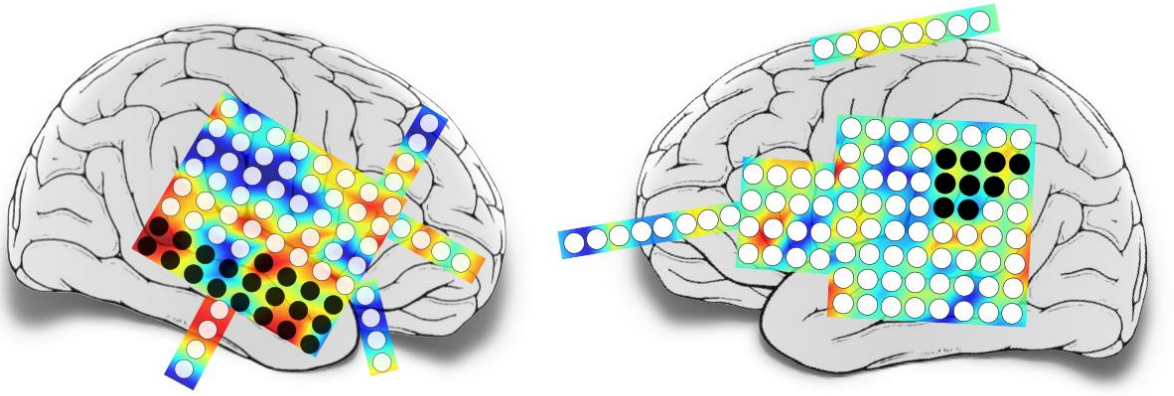

Figure S8.1: Illustration of simulated seizure likelihood for the two exemplary subjects (P1 and P2) when Laplacian montage was applied for pre-processing ECoG signals. In the left panel, for subject P1, the cortical locations with highest seizure likelihood, as depicted by the red areas on the colour plot, are scattered in multiple areas. This is also the case for Subject P2 in the right panel suggesting little to no benefit to use of a laplacian montage.

Laplacian montage refers the signal from an electrode to the signals from its nearest neighbouring electrodes (Lagerlund 2000). An electrode may be surrounded by either four, three or two neighbouring electrodes, depending on whether it is located on the centre, edge or corner of an electrode grid. Similarly, an electrode on the strip will have either two or one neighbouring electrode depending on whether it is located on the middle or at the edge of the strip electrode. In general, let us assume that an electrode channel E has n nearest neighbours denoted by $(E_1, E_2, \ldots, E_n)$. We applied Laplace montage on E to obtain E_l as follows:

$$E_l = E - \frac{1}{n}(E_1 + E_2 + \ldots + E_n)$$

While keeping all other pre-processing steps same as described in Section 2.2, we obtained a functional network from the ECoG signals corresponding to each subject. We incorporated this network in the model and computed simulated seizure likelihood as described in section 2.2 and 2.3. The distribution of simulated seizure likelihood are colour coded in the above figure for the two exemplary subjects P1 and P2.

2. Bipolar Montage

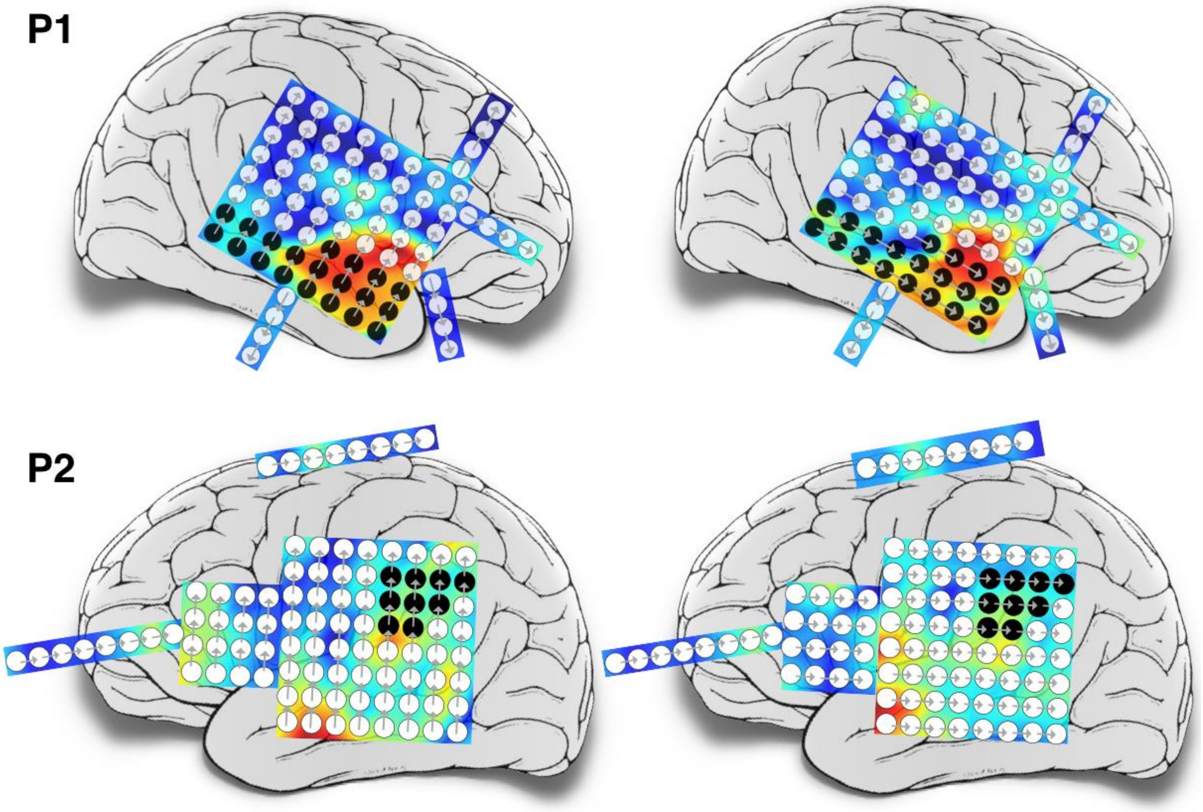

Figure S8.2: Illustration of simulated seizure likelihood for the two exemplary subjects (P1 and P2) when longitudinal and transverse bipolar montage, in the right and left panel respectively, were applied for pre-processing ECoG signals. Note that the regions with highest simulated seizure likelihood, colour coded in red, are in the same cortical locations as in Figure 3. Specifically, for patient P1, the surgical areas diagnosed clinically, correlates with the regions with high simulated seizure likelihood in the right temporal lobe. On the other hand, for patient P2, the regions with highest seizure likelihood are clearly distinct from the clinically diagnosed surgical region in the left parietal cortex.

Bipolar montage approximates the spatial derivative of potential fields by subtracting the potentials measured at adjacent locations (Zaveri et al. 2016). For intracranial electrode grids, the difference between the potential may be obtained by referring each electrode to its adjacent electrode in either longitudinal or transverse direction. For the strip electrodes, bipolar montage can be applied by referring each electrode to its adjacent electrode.

We applied the bipolar reference in both longitudinal and transverse direction which are depicted by the arrows on the electrode grids in Fig. S8.2. We kept all other pre-processing

steps the same as in Section 2.2 and obtained functional network from the ECoG signals. This functional network was incorporated in the model to compute seizure likelihood distribution as detailed in section 2.2 and 2.3. As evident from Fig. S8.2, the cortical areas with high simulated seizure likelihood (shown in red) are in agreement with Figure 3.

Reference:

Lagerlund, T.D., 2000. Manipulating the Magic of Digital EEG: Montage Reformatting and Filtering. *American Journal of Electroneurodiagnostic Technology*, 40(2), pp.121–136.

Zaveri, H.P., Duckrow, R.B. & Spencer, S.S., 2016. On the use of bipolar montages for time-series analysis of intracranial electroencephalograms. *Clinical Neurophysiology*, 117(9), pp.2102–2108.

Supplementary Text S9: Estimating asymmetric network by applying Kullback-Leibler divergence.

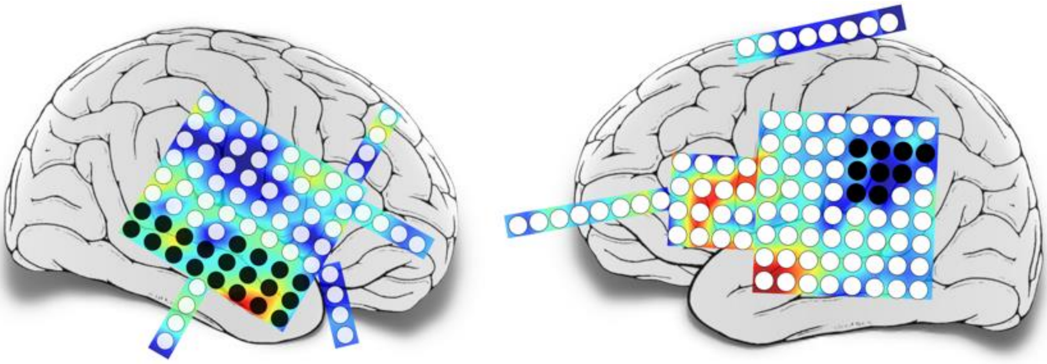

Figure S9. Illustration of simulated seizure likelihood distribution for Subject P1 (on the left) and P2 (on the right) computed by incorporating an asymmetric connectivity parameter in the model. The asymmetric connectivity was obtained from the ECoG signals by applying Kullback-Leibler divergence. Note that the cortical areas with high simulated seizure likelihood (shown in red) are in the same locations as those shown in Fig. 4, where we simulated the model with a symmetric connectivity parameter.

Kullback-Leibler divergence is an information-theoretic interdependence measure, which quantifies the dissimilarity (or distance) between two random variables (or signals). We computed the normalized spectrogram of a signal, $x(n)$, as follows:

$$W_x(n,f) = \frac{|X(n,f)|^2}{\sum_{n,f}|X(n,f)|^2},$$

where, $X(n,f)$ is the Short-time Fourier transform of $x(n)$, obtained using a Hamming window of 1s duration, with 50% overlap between the contiguous sections. The summation in the denominator was carried out over all the time windows and frequency range of 1 to 70Hz. We treated the normalized spectrograms as probability distributions and incorporated them in the Kullback-Leibler divergence equation:

$$K(W_x, W_y) = \sum_{n,f} W_x(n,f) \log \frac{W_x(n,f)}{W_y(n,f)}$$

Kullback-Leibler divergence is an asymmetric measure; it diverges when the distributions are disjoint. Therefore, higher values of $K(W_x, W_y)$ denotes greater dissimilarity (or distance) between the signals and vice-versa. Accordingly, we computed the model connectivity

parameter C, which relates similar signals with higher weights, by obtaining the inverse of K, followed by its normalization between 0 and 1.

Subjects	Actual Outcome	Actual resection vs Random Resection	
		$t_{(act-rand)}$	Cohen's D
**P1**	Good	1.717991	0.163534
**P2**	Bad	-11.605394	-0.270335
**P3**	Good	-3.875874	-1.635309
**P4**	Good	2.453177	0.265004
**P5**	Good	-0.401350	-0.042630
**P6**	Good	11.702740	0.507963
**P7**	Good	-26.264418	-2.527758
**P8**	Good	0.680521	0.200424
**P9**	Good	-4.044922	-0.197963
**P10**	Bad	-0.825498	-0.078635
**P11**	Bad	1.463651	0.082652
**P12**	Bad	11.659090	3.722591
**P13**	Bad	25.742838	1.064707
**P14**	Bad	4.992155	0.359724
**P15**	Bad	-17.463871	-1.214751
**P16**	Bad	12.906590	2.538486

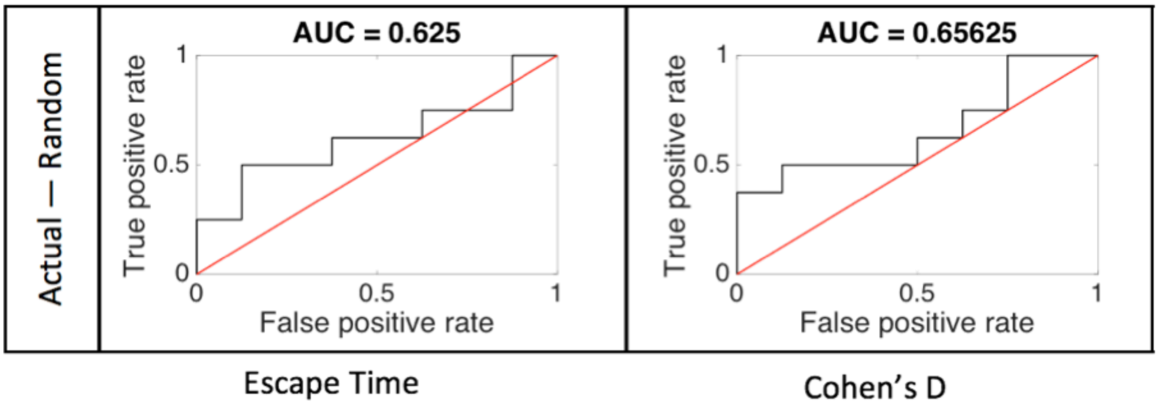

Supplementary Text S10: Alternative network measures.

We consider the elipeptogenicity model (equation 3) as a way of measuring a property of the (functional) network that underpins it, since all other parameters besides the network are homogeneous and the same for all patients. Here we investigate how the model output correlates with other measures of properties of the functional networks. In recent years, graph theory has emerged as a useful way of measuring other properties of brain networks.

Many measures of local (nodal) properties exist and reflect different aspects of the node's role in the network. For example, the **clustering coefficient** (CC) measures the interconnedtedness of the neighbours of a node. This means that nodes with a high clustering coefficient are connected to nodes which are highly (rather than rarely) connected to each other. The **betweenness centrality** (BetC) of a node measures how many times the node occurs in the shortest path between other nodes. Those nodes with a high betweenness centrality are often considered as hubs in the network. Node **strength** (Str) is simply the sum of connection strengths with all other nodes. Nodes with a medium value for strength may be connected to few other nodes very strongly, or many nodes fairly weakly – hence making interpretation more difficult. For this reason, many studies analyse the node degree (simply the number of other nodes which are connected) by arbitrarily thresholding the network to make it sparse. For brevity we do not investigate node degree here though, since the thresholding discards information. Finally, the **eigenvector centrality** (EigC) is another way to determine 'hub' nodes, and is similar to the Google PageRank algorithm. Nodes have a high eigenvector centrality if they are strongly connected to other nodes which are also central to the network (Lohmann *et al.* 2010). Formal descriptions of all of these network measures can be found in (Rubinov & Sporns, 2010; Kaiser, 2011; Lohmann *et al.* 2010). Many other network measures also exist to describe global (as opposed to local) properties of the network. However, we restrict our analysis here to local measures only since we are interested in local properties of the network for surgery localisation. We also limit ourselves to these four measures which have been used primarily in the field before (e.g. Wilke *et al.*, 2011; Hutchings *et al.*, 2015) and an extensive comparison of all measures is beyond the scope of this work.

To investigate the utility of these other measures we used routines implemented in the Brain Connectivity Toolbox in Matlab. We measured the local properties of the nodes in the random-surgery and actual-surgery networks to generate two distributions of each measure, in each subject. The random node surgical distributions, and post-surgical distributions were then compared within each subject to compute the mean change in each each measure (ΔM) for each subject (i), and for each measure (j). To compare the outputs from the different measures we computed Spearman's rank correlation between the different measures of M (Figure S10), and the area under the receiver operating characteristic curve for each measure.

Figure S10 shows the correlations between the changes in measure (either graph-theoretic, or model-derived) across subjects. The matrix is symmetric about the diagonal. Notice the anti-correlation when using clustering coefficient and betweenness centrality in panel a) – this is to be expected since hub nodes tend to have low clustering (also see e.g. Fig 3 in Hutchings *et al.*, 2015). Also notice the anti-correlation when using node strength and the model (top right square of panel a) - this suggests that highly connected nodes have low escape times and hence the removal of them leads to longer escape times overall. The use of clustering coefficient is also highly correlated with both the use of node strength and use of the model, suggesting the origin of the high strength and low escape time may be due to abnormally high interconnectivity within clusters. Overall, inspecting the absolute correlation shows only a partial relationship between each of the graph theoretic metrics and the model – suggesting complementary information may be gained through use of the model, in addition to aiding interpretation in the context of epilepsy.

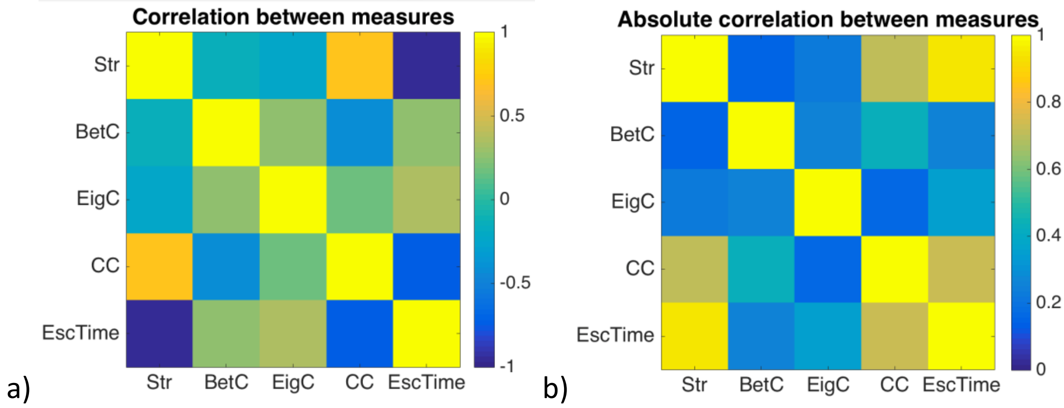

Figure S10. Similarity between mean change following random- and actual-simulated resection using different measures. Changes in different network measures correlate with each other and the model. The changes in clustering coefficient and strength have the highest similarity with the model suggesting the model predictions may be driven by disruptions to highly clustered, strongly connected local networks.

Table S10 shows the AUC of the ROC curve using the different network measures, including our model. Interestingly the clustering coefficient performs very well with an AUC of 0.89. The clustering coefficient is also very strongly correlated with the model predictions. This suggests that this aspect of the network may be driving our positive modelling results. Measures of hubness such as betweenness and eigenvector centrality perform less well, whilst node strength is comparable to the model.

Table S10: Predictions using alternative network measures.

Measure	AUC
CC	0.89
BetC	0.66
Str	0.83
EigC	0.58
Escape Time	0.84

These results using CC suggests hyperconnectivity in local areas (high local clustering), which when disrupted by the removal of highly clustered nodes, may be driving the results we observe. This leads to the intriguing possibility that alternative ways to disrupt high local

clustering in networks may lead to fewer seizures. One way to do this may be through incision (e.g. using multiple subpial transections), rather than complete resection. Surprisingly, the CC has a better predictive value than all other measures, including our model with an AUC of 0.89, as compared to 0.84 for the model. However, this difference amounts to a better classification of two subjects and a misclassification of one subject which was previously correct using the model (this means one better classification overall). Future studies should investigate if this difference is robust and significant for larger datasets and if the CC and Model can be used together to improve prediction. Interestingly, two of the measures of network centrality (BetC, EigC) have less success, suggesting locally clustered subnetworks, as opposed to globally important network hubs drive the genesis of seizures in our model. The good accuracy of strength as a predictor may be reflecting the high interconnectivity of the local clusters. This is in agreement with other modelling studies of spatially constrained spreading in modular networks (see e.g. Kaiser *et al.*, 2007) where in those modular networks, had increased clustering within modules.

Supplementary References

Hutchings F, Han C, Keller S, Weber B, Taylor P, Kaiser M. Predicting Surgery Targets in Temporal Lobe Epilepsy through Structural Connectome Based Simulations. PLoS Computational Biology, 2015.

Kaiser, M. (2011). A tutorial in connectome analysis: topological and spatial features of brain networks. *NeuroImage*, *57*(3), 892–907. http://doi.org/10.1016/j.neuroimage.2011.05.025

Kaiser, M., Görner, M., & Hilgetag, C. C. (2007). Criticality of spreading dynamics in hierarchical cluster networks without inhibition. *New Journal of Physics*, *9*(5), 110–110. http://doi.org/10.1088/1367-2630/9/5/110

Lohmann G, Margulies DS, Horstmann A, Pleger B, Lepsien J, et al. (2010) Eigenvector Centrality Mapping for Analyzing Connectivity Patterns in fMRI Data of the Human Brain. PLoS ONE 5(4): e10232. doi: 10.1371/journal.pone.0010232

Rubinov, M., & Sporns, O. (2010). Complex network measures of brain connectivity: uses and interpretations. *NeuroImage*, *52*(3), 1059–69.

Wilke C, Worrell G, He B. Graph analysis of epileptogenic networks in human partial epilepsy. Epilepsia. 2011;52(1):84–93.
